# Formulation of Sustainable Materials from Agar/Glycerol/Water Gels: An Alternative to Polyurethane Foams in Single-Use Applications

**DOI:** 10.3390/gels11100842

**Published:** 2025-10-21

**Authors:** Perrine Pipart, Bruno Bresson, Alba Marcellan, Théo Merland, Yvette Tran, Jean-Charles Gorges, Olivier Carion, Dominique Hourdet

**Affiliations:** 1 Soft Matter Sciences and Engineering, ESPCI Paris, PSL University, Sorbonne University, CNRS, 75005 Paris, France; perrine.pipart@espci.fr (P.P.); bruno.bresson@espci.fr (B.B.); alba.marcellan@espci.fr (A.M.); theo.merland@espci.fr (T.M.); yvette.tran@espci.fr (Y.T.); 2 IMV Technologies, 61300 Saint Ouen sur Iton, France; jean-charles.gorges@imv-technologies.com (J.-C.G.); olivier.carion@imv-technologies.com (O.C.)

**Keywords:** agar, glycerol, gel, soft material

## Abstract

New compostable materials have been developed to replace single-use soft materials such as polyurethane foams (PUR). To this end, eco-friendly systems have been formulated on the basis of agar gels prepared in mixed solvent (glycerol/water) to meet specifications, i.e., stiffness of several hundred kPa, reasonable extensibility, and good stability when exposed to open air. While the addition of glycerol slows down gelation kinetics, mechanical properties are improved up to a glycerol content of 80 wt%, with enhanced extensibility of the gels while maintaining high Young’s moduli. Swelling analyses of mixed gels, in water or pure glycerol, demonstrate the preservation of an energetic network, with no change in volume, in pure water and the transition towards an entropic network in glycerol related to the partial dissociation of helix bundles. Dimensional and mechanical analysis of gels aged in an open atmosphere at room temperature shows that the hygroscopic character of glycerol enables sufficient water retention to maintain the physical network, with antagonistic effects linked to relative increases in glycerol, which tends to weaken the network, and agar, which on the contrary strengthens it. Complementary analyses carried out on aged agar gels formulated with an initial glycerol/water mass composition of 60/40, the most suitable for the targeted development, enabled the comparison of the properties of agar gels favorably with those of PURs and verified their stability during long-term storage, as well as their non-toxicity and compostability.

## 1. Introduction

Plastics, or synthetic organic polymers, have become essential in everyday life, and their annual global production, which has increased from 2 million tons in 1950 to more than 400 million tons today, is still growing continuously [1,2]. The diversity of their chemical composition and architecture offers an infinity of physical and mechanical properties, paving the way to multiple applications in packaging, transportation, construction, housewares, sports, agriculture. They also allow the conservation and transport of industrial food, medical, and pharmaceutical products in unrivaled hygienic conditions. Difficult to recycle and difficult to digest by microorganisms, they are a source of long-term pollution for the environment when they are produced massively for short-term uses, often less than a year, or for single use. Considered then as waste, they are thrown into landfills, often poorly controlled, or transported to sorting centers where only a small fraction is actually recyclable and recycled [2,3]. Environmental pollution caused by plastic disposal has been recognized as a significant issue [4,5,6], and eco-friendly materials that can substitute conventional plastics in single-use applications are required to mitigate this problem [7]. In this context, bio-sourced polymers issued from renewable resources are the natural alternative, as they generally have biocompatibility and biodegradability characteristics with low impact on the environment.

In the case of the development of soft materials, the properties of polymers can be adjusted by mixing with additives and/or solvents, and for the latter, water is the molecule of choice. The development of three-dimensional networks swollen by water, namely hydrogels, has experienced considerable growth over the last 3 decades due to their versatile use, which covers a wide range of applications, particularly in the cosmetic field [8] as well as in pharmaceutical and biomedical domains [9] with the formulation of increasingly sophisticated flexible materials, able to respond to various stimuli in aqueous environments [10,11,12], and widely used in drug delivery systems [13,14,15] and tissue engineering [16,17]. Nevertheless, hydrogels still have intrinsic weaknesses that limit certain uses or developments on a larger scale. Among others, hydrogels generally display weak mechanical properties related to the low polymer concentration and low viscous dissipation phenomena within the hydrogel [18]. This aspect, which has been the focus of intense research over the last 20 years, has allowed the development of tough hydrogels with mechanical performances sometimes comparable to non-solvated materials. Reinforcement strategies, mainly pioneered in the groups of Gong [19] and Haraguchi [20] in Japan, have considered the creation of sacrificial bonds within the covalent network, either reversible or not, through the design of double networks [19,21] or nanocomposite networks [20,22,23]. Even if this scientific domain has known an exceptional development during the last 20 years using a large set of physical interactions (hydrophobic interactions, electrostatic complexation…) [24,25], their smart design most often integrates synthetic and/or non-biodegradable components, like in the case of polyacrylamide/agar [26] or poly(vinyl alcohol)/agar [27] double networks. Along with the improvement of mechanical properties of hydrogels, the other issue concerns the use of hydrogels in open environments. Indeed, hydrogels are generally developed to perform in aqueous or humid environments, but they rapidly lose their properties and functionalities when exposed to air due to drying [28].

Then, to preserve the initial soft characteristics of swollen networks in open environments, several methods have been considered, such as hydrophobic [28] or elastomeric [29] coatings, which strongly limit and delay the dehydration of hydrogels. Nevertheless, these sophisticated structures are not easy to implement and do not address environmental issues. The best alternative seems, therefore, to replace all or part of the aqueous solvent with non-volatile liquids like ionic liquids [30], deep eutectic solvents [31], or, in a more pragmatic way, using bio-friendly highly concentrated sugar solutions [32], corn syrup [33], or polyol solvents such as glycerol and ethylene glycol [34]. In addition to their biocompatibility, these polyols offer the possibility of retaining the shape and functional properties of soft materials over a wide temperature range due to their low volatility and anti-freezing ability well below zero temperatures [35]. Moreover, as they are highly hygroscopic, they are able to retain significant quantities of water and are used accordingly as emollients in cosmetics in order to maintain skin moisture and hydration [36].

In the strategy of using organic liquid in swollen networks, the organogel can be obtained by immersing the original hydrogel in organic solvent for a while to allow the solvent exchange [37]. This is the case of polyacrylamide/agar organogel prepared by water replacement with glycerol or ethylene glycol for the fabrication of stretchable and stable swollen networks for strain sensing at subzero temperatures [38], as well as agar organogels where water was replaced by organic solvents to develop rigid gels used in paper conservation [39]. More simply, the organogel can also be prepared directly in the polyol formulation [40]. This is typically the case for soft candies (gummies), for instance, which are prepared from gelling agents, corn syrup, and sugar with water contents generally lower than 25 wt% [41]. Gelatin, as well as numerous polysaccharides, has been used as gelling agents to develop flexible and stable materials over time for various applications in the field of food but also for flexible electronics and soft robotics [42,43].

**Figure 1:**
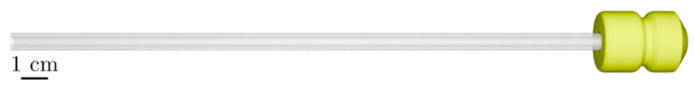
Conventional insemination catheter consisting of a long polypropylene tube onto which an expanded polyurethane foam is molded.

With the aim of replacing single-use soft materials like polyurethane foams used in swine insemination catheters (Figure 1), we develop an eco-friendly approach by investigating soft and stable organogels based on the ternary system agar, glycerol, and water.

**Figure 2:**
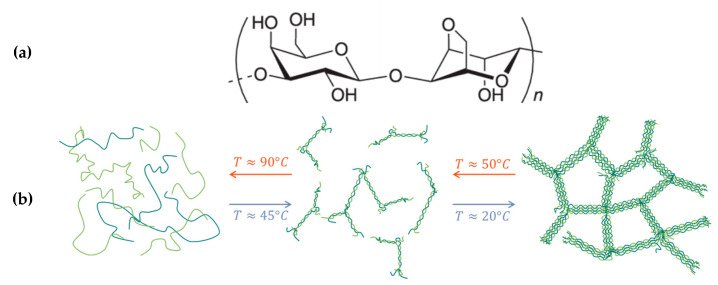
Structure of agarobiose unit (**a**) and schematic representation of gelation upon cooling with double helix formation followed by helix aggregation (**b**).

Agar, also known as agar-agar, is a natural polymer from the galactan family extracted from red seaweeds. It is widely used as a gelling agent in various applications, such as microbiology (bacterial culture and growth), analytical methods with gel separation techniques (electrophoresis), cosmetics (shampoos, creams, lotions), and the food industry (gummies, soups, sauces, yogurts, etc.) [44,45]. Agar is composed of two polysaccharides, agarose and agaropectin, present in varying proportions depending on the origin but with a main fraction of agarose, between 50 and 90% [45]. Agarose is a linear polymer chain made of agarobiose units, a disaccharide alternating between the β-D-galactose and 3,6-anhydro-α-L-galactopyranose units linked in 1–3 and 1–4, respectively (Figure 2). Agaropectin consists of the same agarose backbone substituted with different anionic groups such as sulfate, pyruvate, or glucuronate [46]. Agarose is responsible for the gelation process generated during cooling. It is commonly accepted that agarose gelation is mainly driven by a liquid–liquid-phase separation [44,45,46] with some controversy about the mechanism, either spinodal decomposition [47] or nucleation and growth [48]. It is also recognized that agarose molecules undergo a coil-to-helix [49], or double helix [50], transition upon cooling, leading subsequently to cross-linking and self-aggregation into large bundles (Figure 2) [50].

While the kinetic competition between conformational transition, cross-linking, and liquid–liquid phase separation plays a central role in the overall gelation mechanism, there is a large consensus concerning the general morphology of the gel that can be idealized by a network with large pores and thick walls. This fibrous-like morphology results in (i) “non-swelling” properties in water [51], contrary to common entropic hydrogels; (ii) a high level of rigidity even at very low polymer concentrations; and (iii) a large hysteresis between gelling (below 40 °C) and melting (above 80 °C) points, which makes agar a polymer of choice for the design of stable soft materials. Moreover, agar and agarose are already widely used in biotechnology, and their physical and gelling properties have been extensively studied both in water and organic liquids like polyhydric alcohols [52] or other organic solvents (DMF, DMSO…) [53]. More particularly, in the case of water/glycerol mixtures, it has been shown that the introduction of glycerol up to 80 wt% improved the mechanical properties both in terms of Young’s modulus and extensibility [52]. In the framework of this study, we first investigated the gelling behavior of agar in pure water in order to characterize its intrinsic mechanical properties and structure. In a second step, agar gels were formulated with increasing amounts of glycerol and characterized both in the sol state (rheological behavior) and in the gel state (thermodynamic and mechanical properties). Then the swelling behavior of gels, prepared with various compositions of water/glycerol, was studied in three different environments: water, glycerol, and open air. Finally, given our specifications, the best gel formulation was selected to replace polyurethane foams in swine insemination catheters, and further investigations were carried out on this material to study its toxicity with respect to sperm cells as well as its development on a larger scale with industrial pilot production and complementary tests, including aging, stability and compostability.

## 2. Results and Discussion

### 2.1. Agar Hydrogels

The critical gelation concentration of agar in water (C_gel_), defined as the concentration above which solutions did not flow spontaneously under gravity, was found to be between 0.1 and 0.2 wt% (see Figure S5 in SI). In the present study, mainly agar gels prepared from semi-dilute entangled solutions (C > 1 wt%) have been investigated in order to obtain materials with mechanical properties that can meet the specifications.

**Figure 3:**
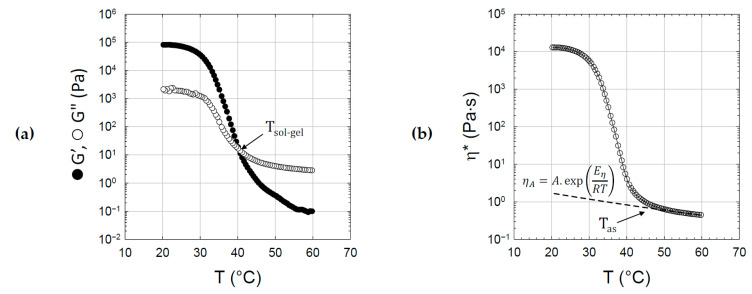
Temperature dependence of viscoelastic properties of an agar formulation prepared at C = 3.6 wt% in water. Variation in elastic (G′ **●**) and viscous (G″ ○) moduli upon cooling and determination of the sol–gel transition temperature (T_gel_) for G′ = G″ (**a**). Variation in the complex viscosity upon cooling and determination of the association temperature (T_as_) from the departure between experimental viscosity (η* ○) and theoretical values (η_A_ ---) extrapolated from the high-temperature behavior using the Andrade relation (**b**).

A typical viscoelastic analysis of agar gelation is given in Figure 3 for an agar solution prepared at C = 3.6 wt% in water. By plotting the dynamic moduli and complex viscosity, three distinct regions can be highlighted:

- Between 60 and 45 °C, the agar solution behaves as a viscous liquid with a viscous modulus (G″) higher than the elastic modulus (G′). The complex viscosity $\eta^{*}$ increases gradually as the temperature decreases according to Andrade’s law $\eta_{A}=A.exp(E_{\eta }/RT)\;$with $E_{\eta }$ the activation energy for the viscous flow. The temperature at which the viscosity diverges from the Andrade relation marks the beginning of the transition: T_as_ ≅ 47 °C.
- Between 45 and 30 °C, the moduli increase sharply with a crossover of G′ and G″ marking the sol/gel transition temperature (T_sol-gel_ ≅ 39 °C), below which the agar formulation exhibits a dominant elastic behavior.
- Below 30 °C, the sample is in the gel state with G′ much higher than G″, and moduli remain practically independent of the frequency. At room temperature, the elastic modulus stabilizes around 80 kPa.

**Figure 4:**
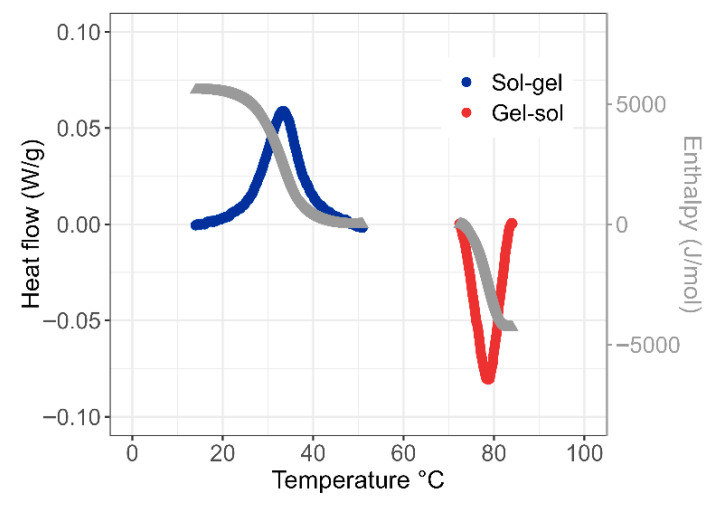
Calorimetric monitoring of the exothermic sol/gel (●) and endothermic gel/sol (●) transitions of agar solution (C = 3.6 wt% in water). The gray curves (●), which are proportional to the integral of calorimetric peaks, give the transition enthalpies, which are around 5 kJ/mol.

Compared to the viscoelastic study, which does not allow the melting of agar gels to be studied as it starts well above 60 °C, the DSC analysis proves to be complementary insofar as it is possible to explore the same transition phenomena over a wider temperature range since the solutions are enclosed in hermetic pans. As shown in Figure 4, the cooling of the agar solution gives rise to an exothermic peak centered at T = 34 °C in good agreement with T_gel_ and the corresponding transition zone depicted previously. The integration of the exothermic peak gives access to a transition enthalpy of 5500 J/mol (or 18 J/g) in good agreement with calorimetric data reported by Holland et al. (ΔH = 20–25 J/g) [54] and Rochas et al. (ΔH =14–20 J/g) [55], who attribute the heat release to the intermolecular cross-linking process rather than to the coil/helix transition. The heating step performed up to 90 °C clearly emphasizes the large hysteresis already described with agar gels [56] with an endothermic melting process that begins around 75 °C. The lower transition enthalpy determined for the melting process, about 4300 J/mol, mainly accounts for the limited duration of the experiment, which stops at T = 90 °C.

**Figure 5:**
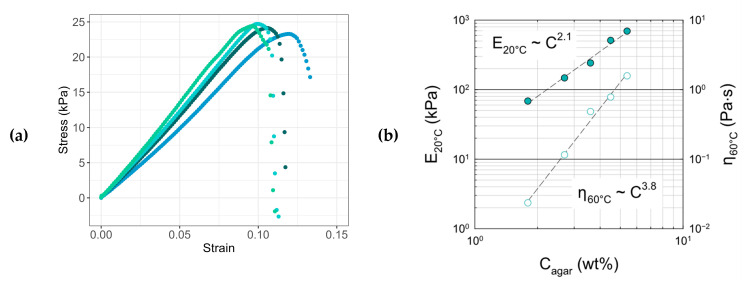
Reproducibility of tensile tests performed at 20 °C on agar gel strips prepared at 3.6 wt% in water (**a**). Variation in the Young modulus at 20 °C (E^20 °C^ ●) and complex viscosity at 60 °C (η^60 °C^ ○) as a function of the concentration of agar in aqueous formulations (**b**).

The viscoelastic analyses were supplemented by mechanical measurements carried out on gel strips stabilized at 20 °C. As shown in Figure 5a, tensile tests display good reproducibility with an average Young’s modulus E = 240 ± 37 kPa and average strain at break ε_R_ = 10 ± 2%. In this case, the approximation E ≅ 3G relating to incompressible materials is well verified.

At 20 °C, in the gel state, the elastic modulus varies in a quadratic manner with the concentration (Figure 5b): $E_{20{}^{\circ}C}\sim G_{20{}^{\circ}C}\sim C^{2.1}$ which is in good agreement with various models beyond the percolation concentration [57] and experimental studies reported in the literature (Mohammed et al. [58]: $G_{20{}^{\circ}C}\sim C^{2.0}$, Lahrech et al. [59]: $G_{20{}^{\circ}C}\sim C^{2.1-2.7}$, and Normand et al. [60]: $G_{20{}^{\circ}C}\sim C^{2.1}$).

Similarly, the concentration dependence of the viscosity determined at 60 °C, $\;\eta_{60{}^{\circ}C}^{*}\sim C^{3.8}$, is in good agreement with similar experiments performed with agar solutions ($\eta_{60{}^{\circ}C}^{*}\sim C^{3.6}$) [59] and with theoretical predictions reported for polymer solutions in the semi-dilute entangled regime: $\eta \sim C^{15/4}$ in good solvent conditions.

**Figure 6:**
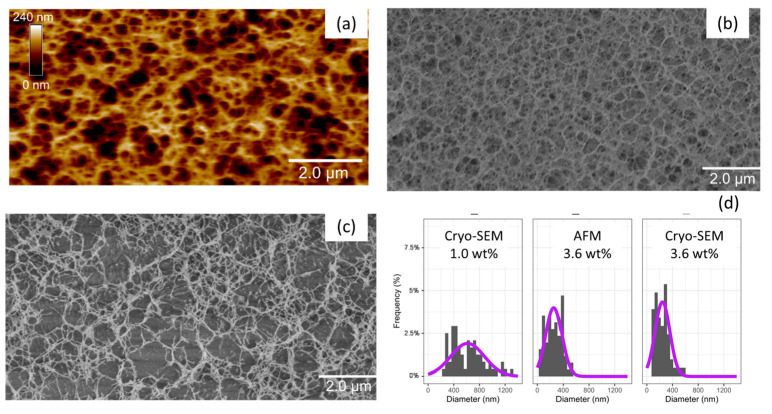
AFM (**a**) and Cryo-SEM (**b**) observations of a 3.6 wt% agar hydrogel (T = 20 °C). Cryo-SEM observation of a 1.0 wt% agar hydrogel (**c**). Histograms of pore size distributions obtained from AFM and Cryo-SEM observations (**d**).

The structure of agar networks was characterized by AFM and cryo-SEM in Figure 6. As reported in the literature, the coil–helix transition of agar chains induced upon cooling, followed by their aggregation in larger bundles, gives rise to a fibrillar structure with large pores and thick walls.

It is interesting to notice that both AFM, performed under water, and SEM, carried out on a cryogenically fixed sample, give similar images for the 3.6 wt% agar gel with similar pore size distributions and mean diameters: d = 293 mm and 305 nm, respectively. The thicker junction areas, identifiable by the white and bright areas on the AFM image, highlight a significant variation in the size of the helix bundles, ranging between a few tens and a few hundreds of nanometers for the thickest fibers. By decreasing the agar concentration from 3.6 wt% to 1 wt%, the fibrous network becomes looser with less regular pore sizes. The 1 wt% agar gel imaged by cryo-SEM shows a wider pore size distribution extending from 200 nm to 1200 nm with an average diameter of 615 nm, in good agreement with data reported in the literature at this concentration (d = 300–800 nm) [61].

The robustness of the fibrous architecture of agar hydrogels can also be highlighted by simple swelling experiments performed in water at room temperature. Indeed, all agar hydrogels initially prepared at different concentrations (${\left(C_{Agar}\right)}_{0}=0.5-3.6\;wt\%)$, maintained their size and shape and kept their initial concentration after 4 days of immersion in water (see Figure S6 in SI). This absence of swelling of hydrogels in water clearly emphasizes the robustness and the dimensional stability of agar networks, even at concentrations as low as 0.5 wt%. This behavior, which originates from the formation of rigid fibers formed by the aggregation of helices during the phase separation process, is very different from that of synthetic networks whose macroscopic swelling mainly depends on the entropy of the constitutive Gaussian chains and solvation conditions. It illustrates one of the specific characteristics of fibrous networks of polysaccharides, which has been widely described in the literature [51,62]. In the case of agar gels, their volume is generally fixed by the concentration of the preparation, which also defines the structure and the porosity of the rigid network during the gelation/phase separation process, as depicted in Figure 6.

### 2.2. Water/Glycerol Agar Gels

**Figure 7:**
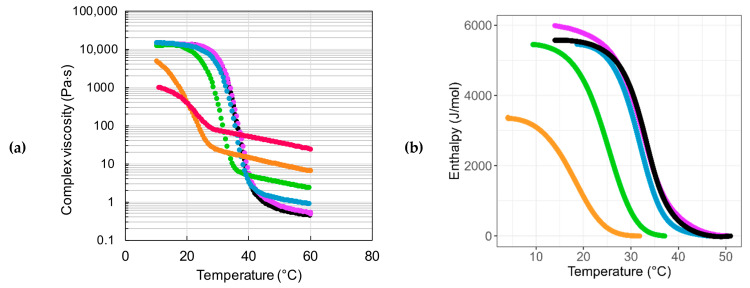
Viscosity (**a**) and enthalpy (**b**) of agar gels as a function of temperature. The agar gels (C_agar_ = 3.6 wt%) were prepared with different weight ratios of glycerol/water (Gx): (● G0, ● G20, ● G40, ● G60, ● G80, ● G100).

In order to study the impact of glycerol in the formation of agar gels, solutions were prepared at a fixed agar concentration (3.6 wt%) using several glycerol/water weight fractions ranging between 0/100 and 100/0. For simplicity, these gels will be called according to the glycerol content in the solvent mixtures (Gx), namely G0, G20, G40, G60, G80, and G100. The rheological and calorimetric profiles of the different formulations are given in Figure 7. As already described in Figure 3 with agar hydrogels, the three different temperature ranges are recovered, with more or less important shifts depending on the glycerol content. At high temperatures, an important increase in viscosity is observed, mainly due to the huge difference between solvent viscosities. At 60 °C, the viscosity of glycerol is almost 175 times higher than that of water, and in this case it is necessary to calculate the specific viscosity of the solutions, $\eta_{sp}=\left(\eta^{*}-\eta_{s}\right)/\eta_{s}$, in order to screen the impact of the solvent viscosity and to emphasize the behavior of the polymer itself in the different formulations.

Moreover, it is also important to consider the volume fraction of agar (ϕ_agar_), instead of its weight concentration or weight fraction, as the densities of water and glycerol are different. The calculated values reported in Table 1 show that the specific viscosity of agar formulations decreases with added glycerol while the volume fraction of agar increases. These variations clearly highlight a decrease in the pervaded volume of the agar chain in the solution state with increasing amounts of glycerol, which therefore behaves as a less effective solvent than water (see Figure S7 and Supporting Information for more details).

The increasing folded conformation of agar with added glycerol can be attributed either to a poorer solvent quality for glycerol compared to water or to a greater flexibility of the agar chain in glycerol due to the breakage of intrachain H-bonds, which participate in the stiffening of the macromolecular structure.

In the intermediate range, between 40 and 30 °C, the transition temperature changes very little for glycerol fractions between 0 and 40 wt%, and then decreases significantly for 60, 80, and 100 wt%. This can be attributed to the increasing viscosity of the medium that slows down the self-assembly of agar. At 20 °C, a stationary behavior seems to be reached for all the gels except for G80 and G100. Whereas the G80 can reach comparable shear modulus levels when stored at 20 °C for one day (exceeding 100 kPa, as we will see in the following with tensile tests), the absence of water molecules, which actively participate in the cohesion of the H-bond network, together with the very high viscosity of glycerol, strongly affect the association process of G100, which will remain very weak even over longer periods. The apparently high value of T_sol-gel_ observed for G100 compared to G80 (see Table S2) is a consequence of the very high viscosity of the solvent, 13 to 22 times higher for G100 compared to G80 between 40 and 20 °C, which proportionally increases the reptation time of entangled polymer chains. The viscoelastic data are in good agreement with the thermodynamic analysis carried out by DSC (Figure 7b) with a good correlation between the association temperatures (see Table S2 in SI for more details). For high glycerol concentration (80 wt%), the large decrease in transition enthalpy from ΔH ≅ 5.5–6 kJ/mol to 3.5 kJ/mol can be related to a lower cohesive energy within the helix bundles due to increased solvation of the chains by glycerol molecules replacing water. No enthalpic transition was observed for G100.

**Figure 8:**
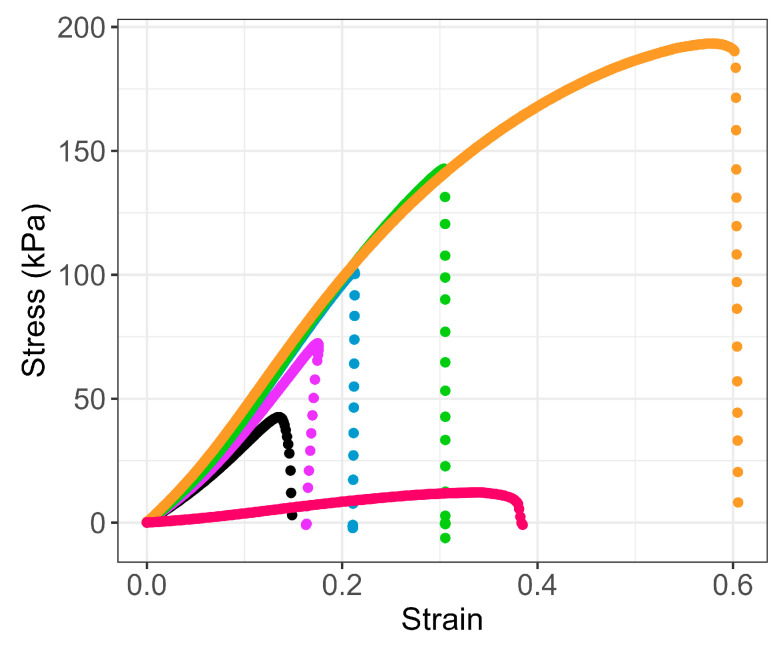
Mechanical behavior at room temperature of agar gels (C_agar_ = 3.6 wt%) prepared with different weight ratios of glycerol/water (Gx): (● G0, ● G20, ● G40, ● G60, ● G80, ● G100).

Finally, at low temperature, the characterization of gels on the rheometer becomes almost qualitative for high glycerol contents (starting at 60 wt%) due to slower gelation kinetics and slight syneresis that precludes accurate computation of stress. In order to obtain a better characterization of agar formulations in the gel state, rheological studies were supplemented by tensile tests performed at room temperature. The stress–strain curves given in Figure 8 clearly show a continuous growth of the elastic modulus together with a strong increase in the strain at break (see Table 2) as the glycerol content rises from 0 to 80 wt%.

In pure glycerol, the gel remains deformable, but its stiffness drops with a maximum stress not exceeding 10 kPa. A similar trend can be found in the literature, where various studies describe the impact of polyhydric alcohols, such as sugars or glycerol, on the mechanical properties of agar gels [52,63]. In this case, it is generally assumed that the addition of such molecules would have the effect of decreasing the size of the junction zones while increasing their number, which could lead to a strengthening of the elastic properties of the gel.

As also mentioned by Normand et al. [64], in the case of agar formulations with sucrose, the increase in viscosity with added sugar could limit the association of helices and the formation of thick bundles. Moreover, by increasing the size of flexible chains linking the junction zones and/or reducing the cohesiveness of these physical crosslinks, the percolating agar network will become less cohesive and subject to greater deformability. The enhancement of the solubility of agar chains to the detriment of helix aggregation is particularly evident in the case of agar formulations in pure glycerol, where the phenomenon of gelation remains very weak, as well as the gels properties.

Nevertheless, for these systems involving large quantities of co-solvent or additives, it is important to consider the agar volume fraction (ϕ_agar_), which is the relevant factor of the formulation, rather than the mass fraction, which is the same for all (3.6 wt%). Under this consideration, Young’s modulus of G/W formulations can be rescaled on the master curve initially established with agar hydrogels (see Figure S8 in SI). This demonstrates that the apparent increase in elastic modulus with glycerol content, from G0 to G80, arises primarily from the relative increase in agar volume fraction. On the other hand, the properties of agar gels formulated in pure glycerol (G100) remain well below the master curve in relation to the formation of a very weak network.

**Figure 9:**
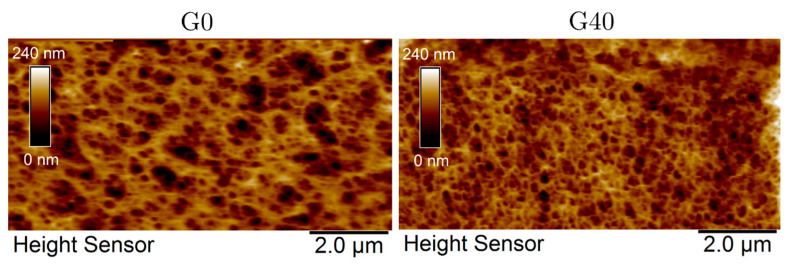
AFM images of agar gels, G0 (**left**) and G40 (**right**), immersed in their own solvent mixture.

In order to probe the structure of the networks, AFM analyses were carried out with various agar gels immersed in their co-solvent. Although agar gels prepared with a high content of glycerol (G60 to G100) were difficult to map due to the high viscosity of the solvent mixture, the good quality of images obtained with gels G0 and G40 allows a quantitative comparison of their structure. Indeed, one can clearly observe in Figure 9 a decrease in the size of the junction zones as well as the pore walls (the fibers in white) with increasing glycerol content (G40 versus G0). In this experiment, the average pore diameter is d = 330 ± 115 nm for G0 against d = 230 ± 36 nm for G40.

By combining the results of mechanics and microscopy, it appears that, like foams or fibrous networks, the elastic modulus measured in the linear regime of agar gels G0 to G80 depends mainly on the volume fraction of polymer constituting the walls, rather than on their structure. On the other hand, the properties at large deformation, and, in particular, maximum extensibility, depend more closely on the structure with the formation of thinner and more extensible fibers with added glycerol.

### 2.3. Composite Agar Gels

In order to improve the properties of the agar gels in relation to the specifications, talc microparticles were added to make the tip less slippery and facilitate its adhesion to the insemination catheter, or even more resistant for applications. For that purpose, a new series of agar gels, Gx^T^, was formulated using the same solvent ratio as before and a fixed concentration of talc (C_Talc_ = 8.3 wt%). From rheological and mechanical analyses (see Figure S9 in SI), the presence of talc does not really influence either the viscosity at high temperature or the phase transition phenomenon. On the other hand, if the mechanical properties of hybrid gels are slightly modified (see Table 2), with an increase in the Young modulus for each formulation, the main reason remains related, as previously, to a slight increase in the agar volume fraction in the hybrid formulations. The main conclusion is the absence of specific interactions between the agar network and talc, which could have strengthened the network. Talc does, however, reduce the slippery nature of the agar gel, which makes it difficult to adhere to the insemination tube.

### 2.4. Stability of Agar Gels

Mostly composed of water and/or glycerol, agar gels can be stored in either closed or open environments, depending on their application. For this reason, the dimensionality of agar gels has been studied in various conditions: either immersed in an excess of solvent (water or glycerol) or exposed to open air. The swelling (or drying) equilibria, as well as mechanical properties, were carried out with all the gels except the one prepared in pure glycerol (G100), which is very fragile and rapidly disintegrates when immersed in an excess of solvent.

#### 2.4.1. Swelling Behavior of Agar Gels in Water

**Figure 10:**
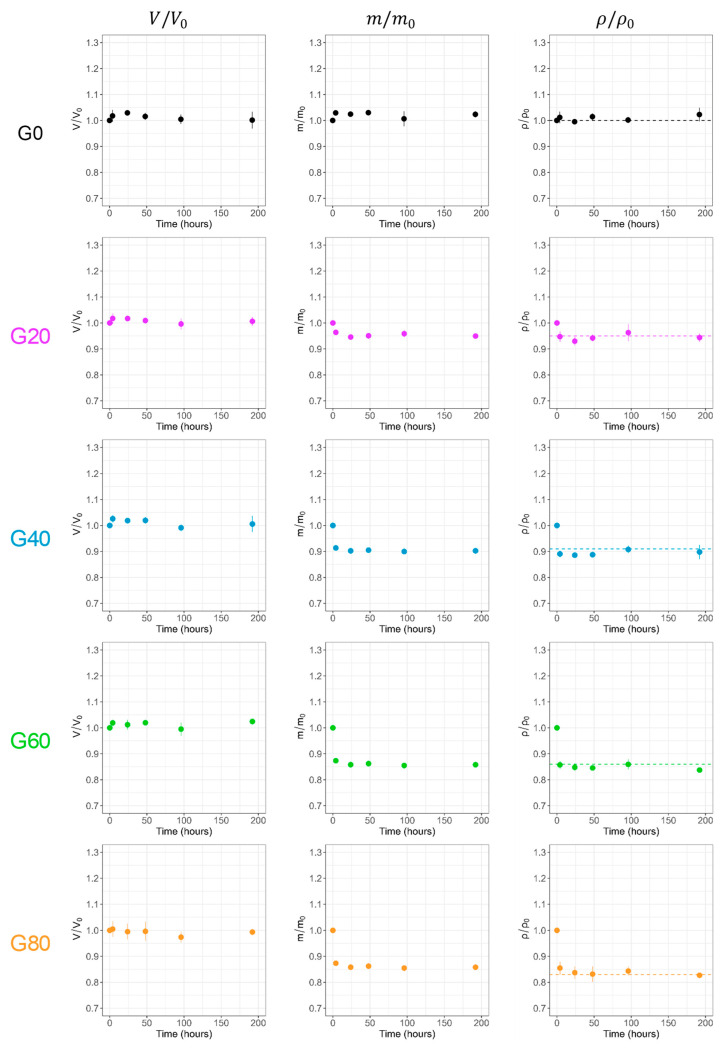
Swelling kinetics of water/glycerol agar gels immersed in water for 8 days with V/V_0_, m/m_0_, and ρ/ρ_0_ the variations in volume, mass, and density. The dotted line represents the theoretical variation in density after complete exchange with water.

The swelling behavior of agar gels prepared at different initial glycerol concentrations has been investigated after immersion in water for 8 days (Figure 10). As described previously with agar hydrogels immersed in water (see Figure S6 in SI), the volume, weight, and density of the hydrogel G0 do not change with time, underlining the robustness and the dimensional stability of the fibrous network in water. Interestingly, what is true for the G0 sample is also observed for all other agar gels, which retain their initial volume throughout the study. Since water and glycerol have different densities, exchanging glycerol for water at constant volume leads to a reduction in gel mass and density, reaching the theoretical value expected for a total glycerol/water exchange after about 4 h. The total removal of glycerol was also confirmed by TGA measurement performed at the end of the study. The main conclusion is that agar networks prepared in glycerol/water mixtures, at least up to 80 wt% glycerol, are therefore sufficiently rigid to retain their volume during solvent exchange.

A complementary study of mechanical properties, carried out after 13 days of exchange in water under isochoric conditions, shows that all the gels exhibit a decrease of 35–40% in elastic modulus compared with their initial state and a very weak deformation at break close to that measured for a fresh G0 hydrogel (see Table 3).

**Figure 11:**
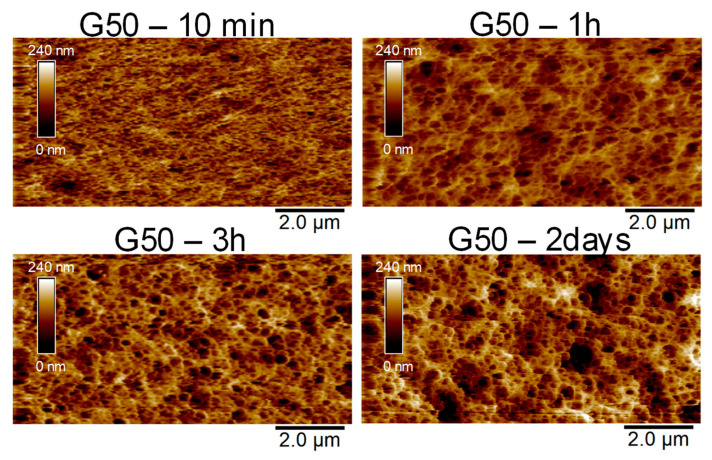
AFM structural analysis of a G50 agar gel immersed in water for 10 min, 1 h, 3 h, and 2 days with regular bath replacement.

In order to obtain more information during solvent exchange, a kinetic microstructural study was carried out by AFM using a G50 agar gel whose formulation corresponds to the maximum glycerol content for which sufficiently accurate imaging can be obtained by this technique. For that purpose, a strip of G50 gel was immersed in water under the AFM tip, and the structure was then imaged at different times (see Figure 11).

At the beginning, after only 10 min of immersion, the structure observed seems rather consistent with a glycerol-containing gel: the junction zones are thin and numerous, and the average pore diameter obtained in this experiment is d_0_ = 162 ± 2 nm. After one hour, the mean diameter increases progressively with d = 299 ± 6 nm and reaches d = 341 ± 30 nm after 2 days, a value close to that initially obtained with a G0 gel. Although the kinetics in the bulk might be slower since the AFM mainly probes the modification of the agar gel surface during solvent exchange, this series of images suggests an increase in aggregation between bundle-forming helices with glycerol removal. This corresponds to a greater phase separation with thicker walls and larger pores. During glycerol/water exchange, the recovery of the structure initially observed with G0 gel (Figure 9), with thick walls and large pores, is in good agreement with the sharp decrease in elongation at break relative to the presence of less deformable fibers. Nevertheless, this structural observation does not justify the decrease in Young’s moduli, which could be attributed to partial hydrolysis and/or release of extractable agar chains during the 13 days of storage in water.

#### 2.4.2. Swelling Behavior of Agar Gels in Glycerol

In contrast to equilibrium data in water, analyses in glycerol are more difficult to perform due to (1) the high viscosity of glycerol, which increases the uncertainty of weighing (difficulty in removing the surface solvent), and (2) the poor mechanical properties of the gels immersed in glycerol, which can also lead to dimensional variations when removed from their baths (see Figure S10 in SI). In order to best interpret the equilibrium data in glycerol, the experimental density variation as a function of time was calculated as before (see details in part 4.6.): $\rho /\rho_{0}=(m/m_{0})/({\left(Lw/L_{0}w_{0}\right)}^{3/2})$ and the volume change was indirectly determined from the mass ratio assuming total exchange of the initial solvent mixture ($\rho_{0}$) with glycerol ($\rho_{Gly}$): $V/V_{0}=(m/m_{0})/(\rho_{Gly}/\rho_{0})$. From the whole set of data (see Figure S11 in SI), it is interesting to note that solvent exchange occurs much more slowly in glycerol, since a decrease in volumes is observed during the first 4 days before the increase thereafter. Despite the dispersion of density data with time, the results suggest total water elimination after 4 to 8 days of equilibrium. This is confirmed by TGA showing that there is no residual water in the G0 hydrogel after 4 days in glycerol (see Figure S12 in SI). This very slow kinetics compared to previous measurements performed in water (t_eq_ < 4 h) can be attributed to a significant decrease in the diffusion coefficients of water and glycerol between the two media considered: the diffusion coefficient of water decreases from 2.3.10^−5^ cm^2^.s^−1^ in water to 1.4.10^−7^ cm^2^.s^−1^ in glycerol, and that of glycerol decreases from 1.0.10^−5^ cm^2^.s^−1^ in water to 2.5.10^−8^ cm^2^.s^−1^ in glycerol [65].

Assuming that all the water has been released after 4 to 8 days, the calculated change in volume V/V_0_ based on this assumption can be considered quantitative beyond this period but remains qualitative for shorter times. Nevertheless, all the studies carried out on the different gels clearly demonstrate an initial deswelling process, corresponding to a much faster outflow of water than the inflow of glycerol, accompanied by a decrease in stiffness and an increase in transparency (see Figure S10 in SI). These variations can be attributed to a gradual dissociation of helix bundles in smaller domains, leading to a relaxation of the stresses locked during gelation. The higher the initial water content, the greater the decrease in volume. After one week of solvent exchange, the re-swelling of the gels over time is a continuation of the previous process with a slow inflow of glycerol into the gel, which seems to reach an equilibrium after 40–50 days. Taking into account the initial volume fraction of agar in the different formulations and the ratio V/V_0_ at the end of the swelling experiments, one can try to calculate the final volume fraction of agar gels in glycerol, which, interestingly, is almost the same and equal to ϕ_agar_ = (4.50 ± 0.15).10^−2^ for all of them. Contrary to gelation in water, which proceeds through double helix formation and phase separation of these helices in robust bundles forming an energetic network, all the gels swollen in glycerol behave as entropic networks able to reach some equilibrium. In these conditions, the disaggregation of bundles releases the constraints initially locked during phase separation. Eventually, dissociated chains could be extracted from the network, but this hypothesis has not been verified. When water, which plays an essential role in the construction of the fibrillar network, has been fully exchanged with glycerol, the organogels become much softer and more deformable, as shown in Table 3 with Young’s modulus and elongation at break measured after 13 days of equilibrium in glycerol.

#### 2.4.3. Aging of Agar Gels in Air

Aging tests were carried out on gel strips left at room temperature and in open air by measuring their dimensions and mass at different times. Except for G0 hydrogel, which is almost completely dehydrated and severely deformed after 48 h (Figure S13 in SI), all the gels prepared in glycerol/water mixtures reach equilibrium after 48 h with a mean residual water content of about 15 wt% (see Figure S14 in SI). Although this study was not carried out under controlled humidity conditions, the relatively large residual water proportion is consistent and would correspond to a relative humidity of the order of 40 wt% with reference to thermodynamic studies carried out on water activity in binary water/glycerol mixtures [66].

**Figure 12:**
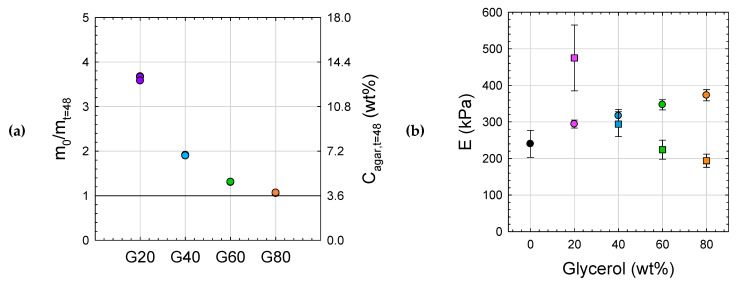
Ratio between the initial mass of the gel ($m_{0}$) and the mass after 48 h aging (m), with ${m_{0}/m=C}_{agar}^{t=48h}/C_{agar}^{t=0}$ (● G0, ● G20, ● G40, ● G60, ● G80) (**a**). Comparison of Young’s moduli of Gx gels before (◯) and after 48 h aging (☐) (**b**).

The water loss during drying results in increased glycerol and agar concentrations, which can be calculated from mass loss assuming that only water is eliminated during aging. As shown in Figure 12a, the higher the initial water content, the higher the agar concentration of the aged samples, with increases of 350% for G20 and 200% for G40 compared to the starting concentration of 3.6 wt%. For G60 and G80 gels, the final agar concentrations are closer to the initial ones, with increases of only 40% (C_eq_ = 5 wt%) and 10% (C_eq_ = 4 wt%), respectively. The direct consequences of air aging are therefore an increase in the concentration of the agar network, which should enhance mechanical properties, and a similar increase in glycerol content up to 80–90% by weight, which would, on the contrary, tend to weaken the network. These antagonistic effects are highlighted in Figure 12b, where the impact of concentration clearly prevails with the gel G20, whose modulus is increased by 50% after 48 h compared to the preparation state. While G40 gel retains its properties, with antagonistic effects offsetting each other, G60 and G80 gels become weaker due to the predominant effect of glycerol, which tends to disaggregate and to soften the helix bundles. The same behaviors are also observed for hybrid gels incorporating talc (see Figure S15 in SI), with increased rigidity for G20^T^, where the increase in agar concentration prevails over glycerol, and lower moduli for G60^T^ and G80^T^, where the high glycerol content outweighs the concentration effect.

Nevertheless, the strengthening by talc, which appeared negligible in the case of fresh formulations, seems to enhance the mechanical properties of aged samples (Table 4).

This could be explained by the increase in talc concentration during aging (almost 28 wt% of talc after 48 h for G20^T^ compared to 8.3 wt% for a fresh gel), which leads to greater hydrodynamic reinforcement. However, for formulations G40^T^ to G80^T^, which do not undergo an important increase in talc volume fraction, one hypothesis would be that, with the partial elimination of water molecules, talc particles could reinforce the network through particle–particle interactions.

### 2.5. Towards Application

Based on this set of results obtained from agar gel formulations in water/glycerol media, we can now move closer to the target application concerning the replacement of catheter tips used in animal insemination. To this end, soft agar gel tips have been molded using fixed concentrations of agar (C = 3.6 wt%) and talc (8.3 wt%) and various weight ratios of glycerol to water.

#### 2.5.1. Aging in Open Air

Aging experiments were carried out on gel tips in similar conditions to those described above for agar strips. Monitoring the mass and height of agar tips as a function of exposure time (see Figure S16 in SI) shows that they reach equilibrium after around 1 week of exposure to open air, with similar deswelling proportional to the initial water content. After 11 days, the mass variations are 88% for G0^T^, 67% for G20^T^, 45% for G40^T^, 28% for G60^T^, and 6% for G80^T^. Similar mass variations were also obtained with accelerated aging tests performed at 30 °C, the equilibrium being reached in only 1 day in these conditions (see Figure S16 in SI).

Although the equilibrium time of gel tips is much longer than gel strips, due to the bigger size of the objects, the comparison between mass and height demonstrates an isotropic deswelling (see Figure S17 in SI) according to ${\left(V_{t}/V_{0}\right)=\left(h_{t}/h_{0}\right)}^{3}=\left(m_{t}/m_{0}\right).\left(\rho_{0}/\rho_{t}\right)$ with V, h, m, and ρ, the volume, height, mass, and density at time 0 or t.

**Figure 13:**
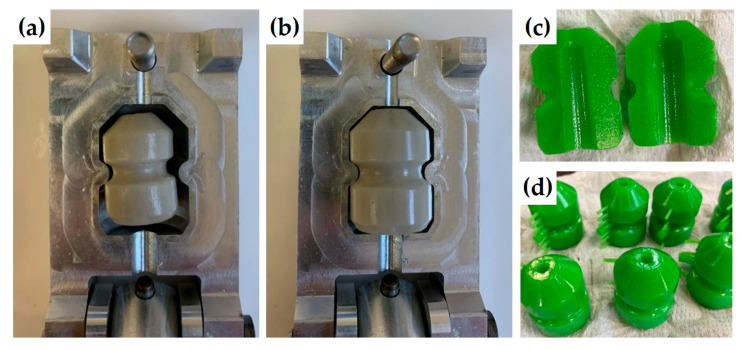
Photos of gels G40^T^ (**a**) and G60^T^ (**b**) in their initial mold after aging 24 h at 30 °C in open air (**c**) and (**d**) processability tests performed at industrial scale with agar/talc formulation G60^T^.

Analysis of these data suggests that the G80^T^ gel, which exhibits the smallest variations in mass and size during open exposure, is best suited to the development of tips with well-controlled dimensions. Nevertheless, its high glycerol content is not relevant for economic reasons, and G60^T^ appears as a better candidate, as its dimensional reduction is only 12%, which remains acceptable for industrial development. By comparison, the gel G40^T^ not only shows a higher decrease in its dimension upon drying (23%) but also a lack of control over the dimensional stability with a warped structure, which does not allow preserving the original shape of the tip (see Figure 13a,b).

Once the formulation has been defined and its feasibility verified for industrial scales (Figure 13c,d), the characteristics of G60^T^ tips were finally studied under conditions close to the application.

#### 2.5.2. Mechanical Properties: G60^T^ Versus PUR

To compare the mechanical properties of agar-talc G60^T^ gel with the polyurethane foam (PUR) currently in use, tensile and compression tests were carried out on materials in strip and tip format, respectively.

##### Tensile Tests

From a general point of view, the mechanical behaviors of the two materials are relatively comparable, although a few differences are worth mentioning. After 48 h of aging at room temperature, the G60^T^ gel has a Young’s modulus of 334 kPa, lower than that of PUR (E = 530 kPa). Nevertheless, while the stress–strain profile of PUR remains linear over the entire deformation range up to failure (see Figure S18 in SI), G60^T^ hardens significantly above 10% deformation. For larger deformations, the trend is reversed, and G60^T^ gel becomes less deformable than PUR. Regarding the elongation at break, ε_R_ = 0.62 for G60^T^ and 0.73 for PUR, we can consider that the two materials are comparable, and this can be a general conclusion over the whole set of tensile properties.

##### Compression Tests

Compression cycles, similar to the sow contractions, have been developed from literature data where authors have assessed the pressure of contractions during estrus at around 7 kPa with an average frequency of about 20 contractions per hour [67,68]. Approximating the surface of the tip to a rectangle of dimension (3 × 2.5) cm^2^, this pressure corresponds to an average force of around 5 N. Based on this information, a series of six compression cycles was performed, applying (1) a compression step at a constant strain rate of 5 mm/min until a force of 5 N was reached, (2) a measure of the displacement during 60 s under a constant force of 5 N, and (3) a decompression step at the same rate of 5 mm/min.

**Figure 14:**
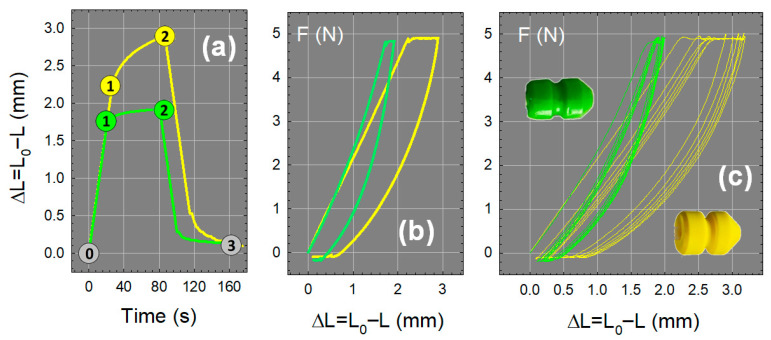
Comparison between compression cycles performed on PUR (yellow ●) and aged G60^T^ tips (green ●). (**a**,**b**): Focus on the first cycle by plotting the displacement versus time and the force versus displacement, respectively. The numbers delimit the phases of the compression cycle: ⓪–① loading from 0 to 5 N at a deformation rate of 5 mm/min, ①–② displacement at constant force (F = 5 N), and ②–③ unloading at the same rate. (**c**) Superposition of the six cycles.

The comparison of the first compression cycle between PUR and G60^T^ tips aged for 48 h (Figure 14a) shows that the G60 is less deformable than PUR, as (1) the corresponding displacement at the end of the compression step (⓪–①) is lower, 1.7 mm against 2.3 mm, and (2) the PUR continues to deform at fixed force (①–②), corresponding to the buckling of cell walls and creep behavior, while the deformation of G60^T^ remains quite stable. At the end of the cycle, the irreversible deformation is around 20% for both materials, with G60^T^ recovering faster than PUR.

The force-displacement analysis of the first cycle (Figure 14b) shows that PUR and G60^T^ behave similarly up to 1.5 N, after which G60^T^ hardens and becomes less deformable, as previously demonstrated in the tensile test. After the first cycle, the next five cycles (Figure 14c) are relatively well superimposed for G60^T^ gel, whereas they progressively shift in the case of PUR, underlining a continuous weakening of mechanical properties during cycling, certainly due to viscoelastic creeping.

From the point of view of the intended application, it is desirable to limit the deformability of the tip. Indeed, when semen is inseminated through the catheter surrounded by the PUR tip, the fluid can escape to the outside (backflow) due to poor positioning or excessive crushing of the probe. The semen is thus rejected outside the uterus, and the use of the less deformable G60^T^ gel could be an additional advantage for the application.

#### 2.5.3. Compostability

**Figure 15:**
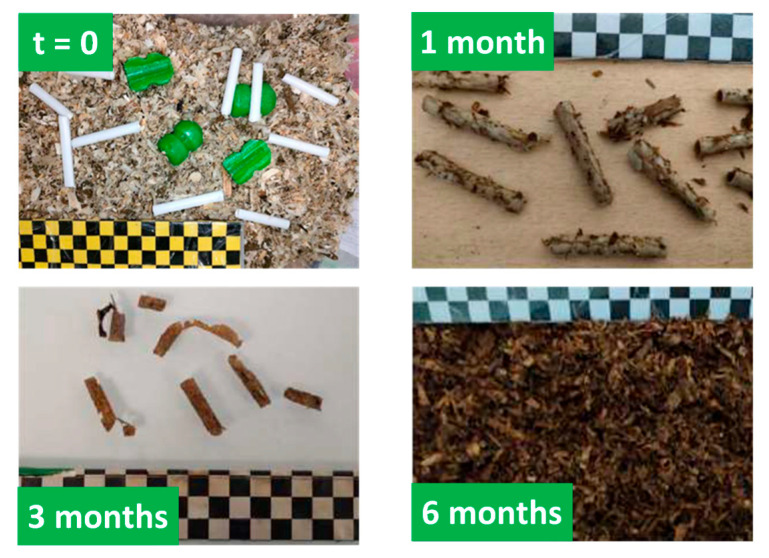
Compostability test carried out on two G60^T^ tips over 6 months.

A compostability test was carried out on the entire catheter made with a paper tube and a G60^T^ tip, with the aim of checking the material’s ability to disintegrate. As shown in Figure 15, the tips were cut into two pieces according to the standard and placed in mature compost integrated into a composting reactor maintained at 20–30 °C. After only 1 month, the tips had completely disappeared from the compost, and only pieces of tubes in the early stages of disintegration were visible. At the end of the trial (6 months), no tube fragments were found in the compost. The complete disintegration of the probe was therefore certified as complying with the existing standard.

#### 2.5.4. In Vitro Toxicity

As described in the experimental section, tip toxicity was assessed by analyzing sperm quality after passage through the insemination probe fitted with a G60^T^ or PUR tip. The average motility of sperm after 1 day or 6 days of storage before the passage through the probe is 88% and 84%, respectively (see Figure S19 in SI). These values drop to 41% and 35%, respectively, after the temperature-resistance test (TRT) performed at 37 °C for 24 h. Under the conditions tested, based on a mixed linear model with fixed effects (analysis time and conditions) and random effects (boars), these results demonstrate that there is no significant impact of the tip material on sperm motility by comparison with the reference PXC.

As with motility, the viability results (see Figure S20 in SI) do not show any significant difference. The viability was around 92% after 1 and 5 days of storage, falling to 80% and 75%, respectively, after the temperature-resistance test. Finally, a more detailed analysis of acrosomal integrity and mitochondrial potential, which allows us to differentiate between viable, viable with intact acrosomes, intact acrosomes, and mitochondrial potential (see Figure S21 in SI), confirms the absence of toxicity of both agar and PUR tips on sperm quality. Indeed, the total proportion of sperm with intact acrosomes was 95% and 90% after 1 and 5 days of storage, respectively, falling to 85% and 75% after TTR. For the population with intact mitochondrial integrity, the proportion was around 55% before and after TTR. This whole set of data confirms that these new materials based on agar, talc, water, and glycerol are non-toxic for sperm.

## 3. Conclusions

As part of the development of new single-use biodegradable materials, this study demonstrates that the addition of glycerol to agar gels improves their properties. The extensibility and stability of agar gel materials in an open atmosphere are enhanced. Among the original points raised in this work, it was shown that the addition of increasing proportions of glycerol made it possible to reduce the size of helix bundles and increase their extensibility without altering their rigidity. In this respect, it is worth highlighting the essential contribution of AFM to the structural analysis of agar gels formulated in mixed solvents, which allowed us to demonstrate variations in the structure of agar networks prepared in water or in the presence of glycerol. It is also interesting to note that all mixed gels prepared with glycerol proportions ranging from 0 to 80 wt% keep their initial dimensions when immersed in pure water. This property, already described in the literature, is associated with the existence of fibrous networks. Moreover, swelling experiments carried out in pure glycerol show much slower exchange kinetics, with the initial departure of water followed by slow diffusion of glycerol into the gel. Partial dissociation of the helix bundles leads in this case to equilibrium swelling properties comparable to those of entropic networks. In aging studies, glycerol enables sufficient water content to be retained to maintain the physical network, with antagonistic effects linked to the relative increases in glycerol, which tends to weaken the network, and agar, which, on the contrary, strengthens it. While the addition of talc to the formulation only slightly alters the mechanical properties of unaged gels, the strengthening of properties after aging suggests the formation of interparticle connections and/or specific interactions between helix bundles and particles. This aspect deserves a more detailed study, not only by extending the range of talc concentrations but also by using other inorganic particles such as silica or clay platelets, which could interact more strongly with the fibrous network. With a glycerol/water ratio of 60/40, the G60^T^ gel offers a good compromise between preservation of mechanical properties with a high Young’s modulus, sufficient extensibility, and limited shrinkage on aging. Complementary analyses carried out with this formulation on molded catheter tips demonstrate the advantage of G60^T^ gel over PUR for cyclic compressions simulating the sow’s vaginal contractions during insemination. Validation of the non-toxicity and biocompostability of these new materials, as well as verification of their industrialization on a pilot scale, confirm their interest for such development and make it possible to envisage the extension of these soft biomaterials to other applications.

## 4. Materials and Methods

### 4.1. Formulations and Gel Formation

Agar-agar sample (E406) was purchased from the company Louis François in Croissy-Beaubourg (France). The water content is about 10 wt%, as determined by thermal gravimetric analysis (TGA). Vegetable-grade glycerol (purity > 99.7 wt%) was obtained from Cargill (Saint-Nazaire, France), and dry talc powder (mean diameter 70 µm) was provided by Provençale S.A. (Brignolles, France). Water (W) was purified with a Millipore system (Merck Millipore, Burlington, MA, USA) combining inverse osmosis membrane (Milli RO) and ion exchange resins (Milli Q), and agar, glycerol (G), and talc (T) were used as received without further purification.

For most of the gels investigated, the agar content was fixed at 3.6 wt%, completed by glycerol/water mixtures prepared with a weight ratio of G/W = 0/100, 20/80, 40/60, 60/40, 80/20, and 100/0. For simplicity, these gels will be called according to the glycerol content in the solvent mixtures (Gx), namely G0, G20, G40, G60, G80, and G100. For complementary experiments, hybrid hydrogels were prepared by adding talc at a weight fraction of 8.3 wt%, while keeping constant the weight fraction of agar at 3.6 wt% and the weight ratio of G/W. They will be called Gx^T^ in the following.

Once powders and solvents have been mixed at room temperature under magnetic stirring, the mixture was heated at 90 °C under stirring in a closed container immersed in an oil bath for 40 min. Preliminary experiments have shown that 40 min was short enough to allow molecular dispersion of agar chains and not too long to avoid thermal degradation (see Figure S1 in SI). After 40 min. at 90 °C, the hot solution was either poured into Petri dishes (120 mm × 120 mm) to obtain gel plates, about 30–40 mL for each dish (thickness ≅ 2–3 mm), or directly injected (about 35 mL) into a special mold designed for preparing soft tips of catheters used in swine insemination (see Figure 1). Finally, Petri dishes and molds were cooled and stored at 4 °C for at least 48 h before experiments.

### 4.2. Linear Rheology

Viscoelastic properties of agar formulations were studied using a stress-controlled rheometer (Haake RS600, Thermo Fisher Scientific, Waltham, MA, USA) equipped with a cone/plate sanded geometry (diameter 35 mm, angle 2°, truncation 103 μm). Dynamic rheology measurements were performed in the linear viscoelastic regime, which was determined for each sample after preliminary stress sweep experiments performed at 60 °C and 10 °C. Particular care was taken to avoid drying of the sample by using a homemade cover, which prevents water evaporation during the experiment. Temperature scanning experiments were performed at constant frequency (1 Hz) and shear stress (1–10 Pa, depending on the sample) by applying cooling scans at 2 °C.min^−1^ between 60 °C and 10 °C with a high-power Peltier system that provided fast and accurate adjustment of the temperature. In these conditions, storage (G’) and loss (G″) moduli as well as complex viscosity (η*) were recorded between 60 °C and 10 °C according to the sample. Reproducibility was verified for each experiment.

### 4.3. Mechanical Testing

Mechanical tests were performed on a standard tensile Instron machine (Instron, Norwood, MA, USA), model 5565, equipped with a 100 N load cell and a video extensometer that follows the local displacement up to 120 mm (with a relative uncertainty of 0.1% at full scale).

#### 4.3.1. Tensile Tests

The samples were cut from gel plates with a punch to the following initial dimensions: L_0_ = 80 mm, w_0_ = 18 mm, and t_0_ = 2 mm (length × width × thickness, see Figure S2 in SI). The initial gauge length was taken as constant (l_0_ ≅ 60 mm). The traction jaws were specifically designed with textured hook-and-loop geometries that allow good grip of gel samples. All tensile experiments were carried out at a strain rate of 0.06 s^−1^, which corresponds to an initial velocity of about 4 mm/s, with at least three tests per sample to check the reproducibility. During the test, force (F) and displacement (l − l_0_), with l_0_ the initial length, were recorded as a function of time while the nominal stress (σ = F/S_0_), with S_0_ the initial cross-section, and the strain (ε = (l − l_0_)/l_0_) were calculated. Note that the video extensometer was used to check that slippage is negligible.

#### 4.3.2. Cyclic Compression Tests

In order to mimic uterine contractions during estrus [67,68], cyclic compression tests were performed on catheter tips. In this case, the initial pipe of the catheter was replaced with a non-deformable brass bar in order to measure only the deformation of the tip. The sample is progressively compressed between two stainless steel surfaces (see Figure S3 in SI) at a speed of 5 mm/min until a force of 5 N is reached. The 5 N compression is then maintained for 60 s before returning to the initial state of zero force at the same speed of 5 mm/min. The compression cycle is repeated six times using the same protocol. As the shape of the nozzle does not allow a precise calculation of the stress (the surface is difficult to approximate), the compression curves will be only presented in terms of force versus displacement for comparison purposes.

### 4.4. Differential Scanning Calorimetry

The gelation and melting processes of agar formulations were investigated by differential scanning calorimetry using a DSC Q200 from TA Instruments (New Castle, DE, USA). Agar gels (about 30 mg) introduced in an aluminum pan and equilibrated with a reference filled with the same quantity of solvent were submitted to the following steps: (1) isotherm at 25 °C for 2 min, (2) heating step up to 90 °C at 2 °C/min, (3) isotherm at 90 °C for 2 min and then (4) cooling step down to −5 °C at 2 °C/min. The transition enthalpy is expressed in joules per mole of disaccharide units (J/mol), using agarobiose as representative repeat units (M = 306 g/mol). All experiments were repeated twice.

### 4.5. Thermal Gravimetric Analysis (TGA)

TGAs were carried out on a thermal analyzer, Discovery SDT-650 (TA Instruments), in order to determine the water, glycerol, talc, and agar contents of the gels over time. Experiments were performed under nitrogen flow using a three-step procedure: (1) heating step up to 100 °C at 20 °C/min, (2) isotherm at 100 °C for 20 min, and (3) heating step up to 1200 °C at 20 °C/min.

### 4.6. Swelling and Drying Experiments

Experiments were performed at room temperature on strip-type gels (V_0_ ≅ 80 × 18 × 2 mm^3^) cut from initial gel plates. The samples (n = 3 for each condition) were weighed (m_0_) and measured with a caliper (length L_0_ and width w_0_) before being immersed in swelling baths, either water or glycerol, which were renewed every day, or exposed to ambient air. For all swelling experiments, gel strips were weighed (m) and measured (length (L) and width (w)) over time during several days after removing the excess solvent with absorbent paper. The relative swelling was calculated either in mass ($m/m_{0}$), or in volume ($V/V_{0}\;=\;{(L.w/L_{0}{.w}_{0})}^{3/2}$), assuming an isotropic swelling of the gels. Similarly, from the above calculations, the variation in gel density during solvent exchange was calculated as follows: $\rho /\rho_{0}\;=\;(m/m_{0})/(V/V_{0})$.

### 4.7. Microscopic Observations

#### 4.7.1. Atomic Force Microscopy (AFM)

Atomic force microscopy experiments were performed with a Dimension ICON™ from Bruker^®^ associated with a Nanoscope 5 controller (Bruker, Billerica, MA, USA). The observations were carried out using an MLCT tip with a stiffness of 0.01 N/m in immersion and contact mode. As the gel adhered to the edges of the container, no glue was used to fix the sample, which was covered by a thin layer of the selected solvent.

#### 4.7.2. Scanning Electron Cryomicroscopy (Cryo-SEM)

Structural analysis of agar hydrogels was also investigated at ICS Strasbourg by scanning electron cryomicroscopy. Experimentally, a piece of hydrogel was placed in a cryogenic sample holder and quickly dipped into liquid ethane, which instantly freezes the sample with the formation of amorphous ice. Subsequently, the sample was transferred into the Quorum PT 3010 chamber attached to the microscope. The solidified sample is then fractured with a razor blade and etched at −90 °C (sputter coated with platinum) before being transferred into the FEG-cryoSEM (Hitachi SU8010, Hitachi, Tokyo, Japan) and observed at 1 kV and −150 °C.

### 4.8. Compostability

The compostability of swine insemination catheter was carried out at the OSE chemical analysis laboratory (Annecy, France). The domestic compostability of a given material is delivered according to the OK compost HOME program by the company TUV AUSTRIA (European standard EN 13432:2000/AC:2005) [69]. Four main characteristics are considered: (1) biochemical composition of the product, (2) biodegradability, (3) disintegration applied at temperatures of 20–30 °C, and (4) effect on compost quality after disintegration (known as phytotoxicity). As required by the standard, the whole catheter designed with a paper tube, replacing polypropylene, and G60 tip was cut into two pieces and placed in mature compost* at 20–30 °C integrated into a watertight 5 L container with two holes for passive aeration.

* A mature compost has a uniform and homogeneous texture that resembles potting soil in which dry and green organic waste have completely decomposed.

### 4.9. In Vitro Toxicity

Trials were carried out to test the toxicity of biodegradable tips for spermatozoa. In order to represent the best conditions in which the tool is used, it was considered that the probe is only an object through which the semen passes. It is not intended to remain in contact with the sperm cells for longer than the time required for insemination. Moreover, the tip is in contact with the semen only on the final part of the object and not along its entire length (see Figure S4 in SI). To this end, a toxicity test protocol fairly representative of reality was set up and detailed in the supporting information.

## 5. Patents

A French patent application has been filed for the composition of the tip, under the filing number FR2401479.

## Figures and Tables

**Table 1:** Volume fraction (ϕ_agar_) and specific viscosity (η_sp_) at 60 °C of formulations Gx prepared at fixed agar concentration (C = 3.6 wt%) (see Table S1 in SI for detailed calculation).

Sample	G0	G20	G40	G60	G80	G100
ϕ_agar_	0.035	0.037	0.039	0.041	0.043	0.045
η_sp_	930 ± 360	690 ± 145	685 ± 125	740 ± 140	590 ± 120	290 ± 6

**Table 2:** Comparison of mechanical properties at room temperature of agar gels (C = 3.6 wt%) prepared in mixed solvent (glycerol/water) without or with added talc particles (C_talc_ = 8.3 wt%).

Gel	Without Talc				With Talc		
	ϕ_agar_	G’ (kPa)	E (kPa)	ε_R_	ϕ_agar_	E (kPa)	ε_R_
G0	0.035	81	240 ± 37	0.10 ± 0.02	0.0380	283 ±12	0.10 ± 0.02
G20	0.037	93	294 ± 11	0.13 ± 0.04	0.040	399 ± 81	0.13 ± 0.02
G40	0.039	103	317 ± 17	0.18 ± 0.03	0.0415	368 ± 93	0.13 ± 0.01
G60	0.041	80	347 ± 14	0.27 ± 0.03	0.0434	443 ± 36	0.22 ± 0.06
G80	0.043	46	373 ± 15	0.44 ± 0.08	0.0453	392 ± 85	0.31 ± 0.10
G100	0.045	10	27 ± 5	0.29 ± 0.06			

**Table 3:** Young’s modulus (E) and elongation at break (ε_R_) of agar gels in the preparation state and after immersion for 13 days in water or in glycerol.

Gel	Preparation State		After 13 Days in H_2_O		After 13 Days in Glycerol	
	E_0_ (kPa)	ε_R_	E (kPa)	ε_R_	E (kPa)	ε_R_
G0	240 ± 37	0.10 ± 0.02	148 ± 7	0.08 ± 0.05	51 ± 16	0.82 ± 0.04
G40	317 ± 17	0.18 ± 0.03	205 ± 48	0.11 ± 0.03	56 ± 18	0.64 ± 0.29
G60	347 ± 14	0.27 ± 0.03	221 ± 21	0.08 ± 0.02	44 ± 8	0.66 ± 0.16
G80	373 ± 15	0.44 ± 0.08	226 ± 35	0.06 ± 0.02	35 ± 4	0.61 ± 0.17

**Table 4:** Comparison of mechanical properties after aging 48 h at room temperature of agar gels (C = 3.6 wt%) without (Gx) or with (Gx^T^) added talc particles (C_talc_ = 8.3 wt%).

Gx	Without Talc		Gx^T^	With Talc	
	E (kPa)	ε_R_		E (kPa)	ɛ_R_
G20	475 ± 90	0.58	G20^T^	1862 ± 126	0.81
G40	294 ± 34	0.45	G40^T^	477 ± 66	0.63
G60	224 ± 26	0.30	G60 ^T^	334 ± 9	0.62
G80	194 ± 18	0.30	G80^T^	301 ± 35	0.41

## Data Availability

The data presented in this study are available on request from the corresponding author due to patent filing.

## Supplementary Materials

The following supporting information can be downloaded at: https://www.mdpi.com/article/10.3390/gels11100842/s1, Figure S1: Temperature dependence of complex viscosity during cooling. Figure S2: Schematic representation of agar strip used for tensile test. Figure S3: Cyclic compression test performed on designed gel tips. Figure S4: Schematic representation of materials used for semen collection. Figure S5: Pictures of agar gel formation versus initial concentration. Figure S6: Agar gel concentration at equilibrium versus their preparation state. Table S1: Volume fraction and specific viscosity at 60 °C of formulations Gx prepared at fixed agar concentration (C = 3.6 wt%). Figure S7: Variation in the specific viscosity of agar solutions at 60 °C as a function of agar volume fraction in Gx formulations. Table S2: Characteristic temperatures of association and gelation upon cooling as determined by DSC and rheology. Figure S8: Variation in Young’s modulus as a function of the agar volume fraction in the Gx formulations without and with talc. Figure S9: Rheological and mechanical analyses performed on composite agar gels. Figure S10: Photos of G0 and G20 gels before and after immersion in glycerol. Figure S11: Swelling kinetics of glycerol/water agar gels immersed in glycerol for 45 days. Figure S12: TGA monitoring of G0 gel during swelling in glycerol. Figure S13: Images of G0 hydrogels before and after aging at room temperature and in open air. Figure S14: Kinetics of drying of Gx gels during aging at room temperature and in open air and TGA after 48 h of aging. Figure S15: Comparison of Young’s moduli of Gx^T^ gels before and after 48 h aging. Figure S16: Kinetics of drying in open air and room temperature of Gx^T^ gels. Figure S17: Comparison between height and mass analyses performed on agar gel tips after 11 days of drying. Figure S18: Comparative stress–strain curve obtained with PUR and G60^T^ agar gel after aging 48 h at room temperature. Figures S19–S21: Evaluation of sperm motility, viability, and acrosomal and mitochondrial integrity after 1 or 6 days of storage at 17 °C and passage of the semen through the probe [70].
