# Supplementary material for: Formulation of Sustainable Materials from Agar/Glycerol/Water Gels: An Alternative to Polyurethane Foams in Single-Use Applications
Source: Gels. 2025 Oct 21;11(10):842. doi: 10.3390/gels11100842 (PMC12564599; doi:10.3390/gels11100842)
Supplement: Supplementary file 1 [file gels-11-00842-s001.zip › gels-3921595-supplementary.pdf]

# *Supplementary information*

# Formulation of Sustainable Materials from Agar/Glycerol/Water Gels: An Alternative to Polyurethane Foams in Single-Use Applications

*Perrine Pipart [1,2], Bruno Bresson [1], Alba Marcellan [1], Theo Merland [1],*

*Yvette Tran [1], Jean-Charles Gorges [2], Olivier Carion [2] and Dominique Hourdet [1]*

[1]Soft Matter Sciences and Engineering, ESPCI Paris, PSL University,

Sorbonne University, CNRS, F-75005 Paris, France

[2]IMV Technologies, 61300 Saint Ouen sur Iton, France

## 1. Formulation and gelation

Agar formulations were prepared by first mixing agar powder and solvents at room temperature under magnetic stirring, before being heated to 90 °C under stirring in a closed vessel immersed in an oil bath. At this temperature, mixing time is a key parameter to avoid hydrolysis of polysaccharide chains and preliminary gelation experiments have been carried out to investigate the viscoelastic properties as a function of mixing time at 90 °C. As shown in **Figure S1**, all the formulations prepared between 40 and 270 minutes exhibit the same viscosity in the sol state at 60 °C ($\eta^* = \eta_0$) as well in the gel state at 20 °C ($\eta^* = G'/\omega$) and the same transition temperature. Only the formulation prepared during 330 minutes evidences a slight decrease of viscoelastic properties properties as well as a shift of the sol/gel transition to a lower temperature which underlines a possible degradation of the chains during preparation. Based on these results, a mixing time of 40 minutes at 90 °C has been applied to all agar formulations after which, the hot solution was either poured into Petri dishes (120 mm x 120 mm) to obtain gel plates, approx. 30-40 mL for each dish (thickness ≅ 2-3 mm), or directly injected (approx..

35 mL) into a special mold designed to prepare the soft tip of catheters used in swine insemination. Finally, Petri dishes and molds were cooled and stored at 4 °C for at least 48 hours prior to the experiments.

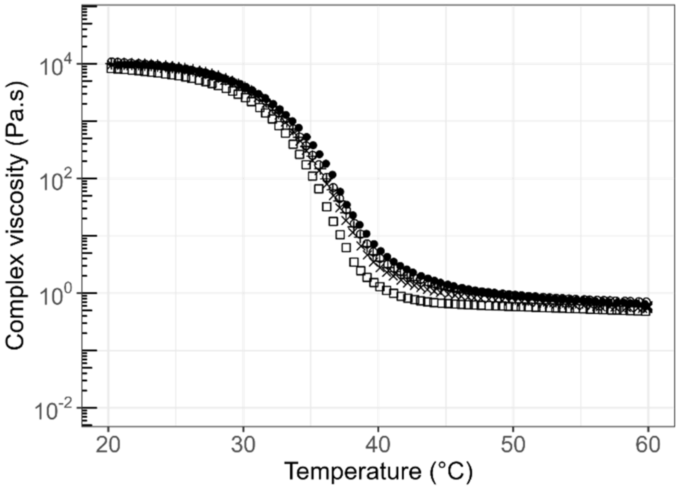


**Figure S1**. Temperature dependence of complex viscosity during cooling for the same formulation of agar (C = 3.6 wt% in water) prepared with different solubilization times at 85 °C:
t = 0h40 (●); 1h30 (○); 2h30 (+); 4h30 (×); 5h30 (□)

## 2. Mechanical testing

Mechanical tests were performed on a standard tensile Instron machine, model 5565, equipped with a 100 N load cell and a video extensometer which follows the local displacement up to 120 mm (with a relative uncertainty of 0.1 % at full scale).

### *2.1. Tensile tests*

The samples were cut from gel plates with a punch to the following initial dimensions: $L_0$ = 80 mm, $w_0$ = 18 mm, and $t_0$ = 2 mm (Length x width x thickness, see **Figure S2**). The initial gauge length was taken constant ($l_0 \cong 60$ mm). The traction jaws were specifically designed with textured hook-and-loop geometries that allow good grip of gel samples. All tensile experiments were carried out at a strain rate of 0.06 $s^{-1}$, which corresponds to an initial velocity of about 4 mm/s, with at least three tests per sample to check the reproducibility. During the test, force ($F$) and displacement ($l$-$l_0$), with $l_0$ the initial length, were recorded as a function of time while the nominal stress ($\sigma = F/S_0$), with $S_0$

the initial cross-section, and the strain ($\varepsilon = (l-l_0)/l_0$) were calculated. Note that the video extensometer was used to check that slippage is negligible.

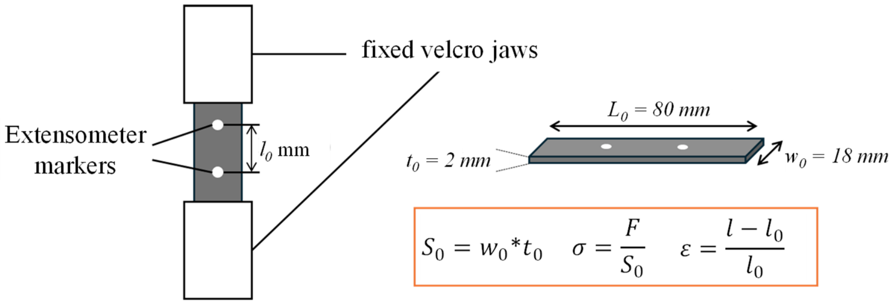


**Figure S2**. Schematic representation of agar strip (length $L_0$, width $w_0$ and thickness $t_0$) used for tensile test, with σ and ε the stress and strain, F the tensile force, $S_0$ the initial cross-section, $l_0$ the initial length between markers and $l$ the length during deformation as determined by the extensometer.

*2.2. Cyclic compression tests*

In order to mimic uterine contractions during estrus (1-2), cyclic compression tests were performed on catheters tips. In this case, the initial pipe of the catheter was replaced with a non-deformable brass bar in order to measure only the deformation of the tip. The sample is progressively compressed between two stainless steel surfaces (**Figure S3**) at a speed of 5 mm/min until a force of 5 N is reached.

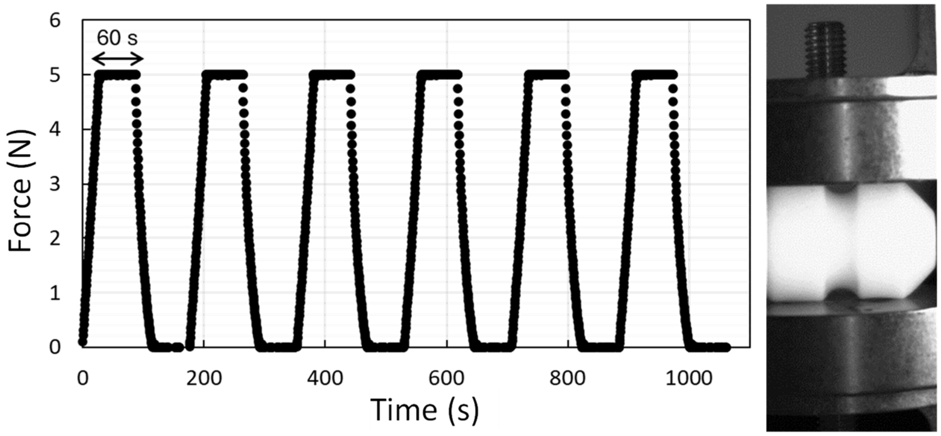


**Figure S3.** Cyclic compression test performed on designed gel tips.

The 5 N compression is then maintained for 60 s before returning to the initial state of zero force at the same speed of 5 mm/min. The compression cycle is repeated 6 times using the same protocol. As the shape of the nozzle does not allow a precise calculation of the stress (the surface is difficult to

approximate), the compression curves will be only presented in terms of force versus displacement for comparison purposes.

### 3. In vitro toxicity analysis

Trials were carried out to test the toxicity of biodegradable tips for spermatozoa. In order to represent the best conditions in which the tool is used, it was considered that the probe is only an object through which the semen passes. It is not intended to remain in contact with the sperm cells for longer than the time required for insemination. Moreover, the tip is in contact with the semen only on the final part of the object and not along its entire length (see **Figure S4**).

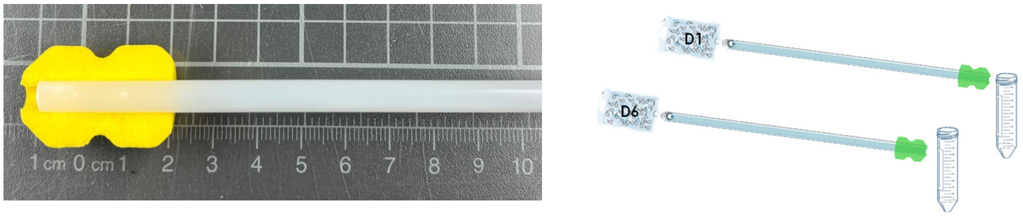


**Figure S4. Left:** Position of the tube in the tip. **Right**: Schematic representation of how the bags are connected to the catheter and how the semen is collected in the tubes.

To this end, a toxicity test protocol fairly representative of reality was set up. Semen previously diluted to a concentration of $35.10^6$ spz/mL was packaged in bags and stored either for 24 hours or for 5 days at 17 °C. These two conditions were devised with the aim of ageing or not the semen in the bag used by customers in order to represent cases where insemination does not take place exactly on the day of dilution but rather a few days later. After 24 h or 6 days, the bags were connected to a polypropylene tube (the material currently used), which is itself fitted with a gel G60 tip or a PUR tip. The semen then passes through the probe over a total period of around 5 minutes (at a moderate flow rate to account for long inseminations) and is collected and stored in 50 mL tubes (**Figure S4**). After passage through the catheter, a first analysis of the semen was carried out (labelled D1 or D6 according to the storage time in the bag) followed by a second one for heat resistance performed after storage in the tube for 24 hours at 37 °C for 24 hours (namely D2 or D7). Two replicas of the gel tip (named G60-1 and G60-2) and one PUR tip were tested in parallel with a control condition (named PXC, for PrimXcell, a control medium supplied by IMV Technologies). Finally, the mobility and

viability of the sperm were characterised using the integrated visual optical system (IVOS®). The mitochondrial potential and acrosome integrity were evaluated using flow cytometry (EasyCyte™). Briefly, for mitopotential, 2 µL of JC-1 fluorophore diluted at 0.05 mg/mL in DMSO were added in each well containing 200 µL of EasyBuffer A™ (EBA). Then 60 000 sperm cells were added in each well and the well plate was incubated during 30 minutes at 37 °C before analyses. To assess the acrosome integrity of sperm cells, EasyKit 5™ was used. Pellets were resuspended using 200 µL of EBA in each well, 60 000 cells were then added and the plate was incubated 15 minutes at 37 °C before analyses. To increase the accuracy of toxicity tests, three series of boars were used in order to increase the biological variability ($n_1 = 8$, $n_2 = 8$, $n_3 = 7$, $n_{tot} = 23$ boars) and all the analyses were duplicated for each boar and each condition.

**4. Agar hydrogels**

The critical gelation concentration of agar in water ($C_{gel}$), defined as the concentration above which solutions did not flow spontaneously under gravity, was found to be between 0.1 and 0.2 wt% (**Figure S5**).

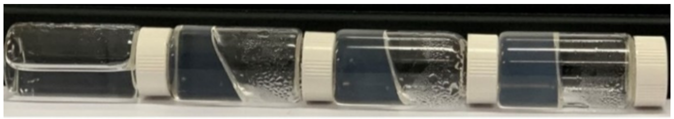


**Figure S5**. Pictures of aqueous agar formulations initially prepared at 90 °C before being cooled at 20 °C. The critical gelation concentration of agar ($C_{gel}$) is estimated between 0.1 wt% (liquid behaviour) and 0.2 wt% where a weak percolated network is formed.

Above $C_{gel}$, agar hydrogels form rigid fibrous networks which originate from the aggregation of helices during the phase separation process induced by cooling. This peculiar morphology, which is very different from that of entropic networks built from Gaussian chains, is responsible for the robustness and the dimensional stability of agar networks immersed in water, even at concentration as low as 0.5 wt%. Such behaviour is highlighted in **Figure S6** where all agar hydrogels, initially prepared at different concentrations ($(C_{Agar})_0 = 0.5 - 3.6\ wt\%$), maintained their size and shape and keep their initial concentration after 4 days immersion in water at room temperature.

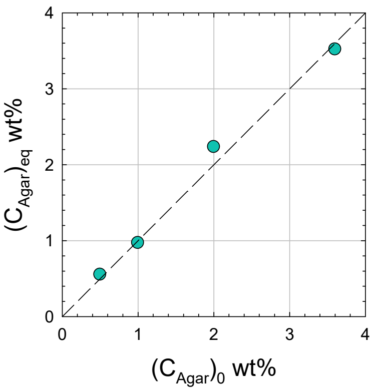


**Figure S6**.

Variation of the concentration of agar gel after immersion during 4 days in water at room temperature $(C_{Agar})_{eq}$, versus their initial concentration in the preparation state $(C_{Agar})_0$.

**5. Specific viscosity of agar in solvent mixtures**

At high temperatures, an important increase in viscosity is observed, mainly due to the huge difference between solvent viscosities. At 60°C, the viscosity of glycerol is almost 175 times higher than that of water and in this case it is interesting to calculate the specific viscosity of the solutions, $\eta_{sp} = (\eta^* - \eta_s)/\eta_s$, in order to screen the impact of the solvent viscosity and to emphasize the behaviour of the polymer itself in the different formulations. The calculated values given in **Table S1** show that the specific viscosity of agar formulations decreases with added glycerol. Moreover, as the volume fraction of agar (ϕ) regularly increases in the formulations, from G0 to G100, the overall variation of $\eta_{sp}$ clearly highlight a decrease of the pervaded volume of the agar in the sol state ($V_{agar,x}$) with increasing weight fraction, x, of glycerol. A qualitative analysis can be carried out by assuming that the same master curve obtained in water as a good solvent can be used to describe the variation in the specific viscosity of agar in glycerol/water formulations, $\eta_{sp} \sim (\phi/\phi^*)^{3.8}$, with $\phi^*$ the overlap volume fraction of agar chains.

**Table S1**. Volume fraction ($\phi_{agar}^{60°C}$) and specific viscosity ($\eta_{sp}^{60°C}$) at 60 °C of formulations Gx prepared at fixed agar concentration (C=3.6 wt%). The volume fraction of agar was calculated as follows from the weight composition of the formulation using the density of the solvent mixture ($\rho_{mixture}^{60°C}$) (3) and assuming $\rho_{agar}^{60°C} = 1.0\ g/mL$ (4) : $\phi_{agar}^{60°C} = 3.6/\left(3.6 + 96.4/\rho_{mixture}^{60°C}\right)$

| Sample | G0 | G20 | G40 | G60 | G80 | G100 |
|---|---|---|---|---|---|---|
| $\rho_{mixture}^{60°C}$ (g/mL) | 0.98 | 1.03 | 1.08 | 1.13 | 1.19 | 1.24 |
| $\phi_{agar}^{60°C}$ | 0.035 | 0.037 | 0.039 | 0.041 | 0.043 | 0.045 |
| $\eta_{sp}^{60°C}$ | 930±360 | 690±145 | 685±125 | 740±140 | 590±120 | 290±6 |

As shown in **Figure S7**, where the scaling relation $\eta_{sp}{\sim}\phi^{3.8}$ has been plotted for aqueous solutions of agar, the relative ratio of the overlap concentration of Gx formulations ($\phi_w^*/\phi_x^*$) can be extrapolated by translating the specific viscosity of Gx onto the G0 scaling relation (see example for G100 in **Figure S7**).

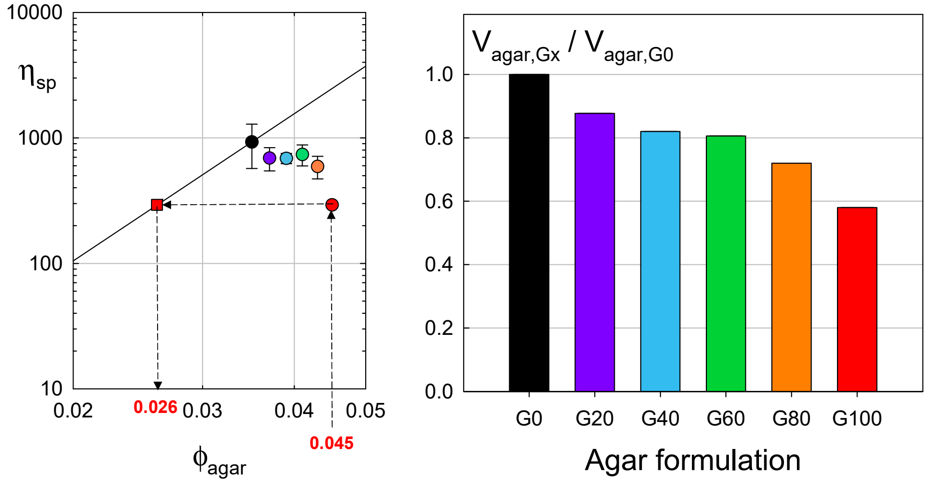


**Figure S7. Left:** Variation of the specific viscosity ($\boldsymbol{\eta_{sp}} = (\boldsymbol{\eta^*} - \boldsymbol{\eta_s})/\boldsymbol{\eta_s}$) of agar solutions at 60 °C as a function of agar volume fraction in Gx formulations: (● G0, ● G20, ● G40, ● G60, ● G80, ● G100). The solid line corresponds to the scaling relation obtained at 60 °C with agar formulations in water ($\boldsymbol{\eta_{sp}} = \mathbf{K}\boldsymbol{\phi}^{3.8}$). The dotted lines exemplify for G100, how the corresponding volume fraction in water ($\phi_2$=0.026 ■) is calculated from $\boldsymbol{\eta_{sp}}$ experimentally determined at $\phi_1$=0.045 (●); with $\boldsymbol{\phi_1}/\boldsymbol{\phi_2} = \boldsymbol{\phi_w^*}/\boldsymbol{\phi_x^*}$. **Right**: Variation of the volume of the agar chain in formulation Gx ($V_{agar,Gx}$) relative to G0 ($V_{agar,G0}$).

In the same **Figure S7**, the bar chart of $V_{agar,x}/V_{agar,w}{\sim}\phi_w^*/\phi_x^*$ highlights a continuous deswelling of the pervaded volume of agar chain with increasing fraction of glycerol. This can be attributed either to a poorer quality of solvation of glycerol compared to water, or to a greater flexibility of the agar chain in glycerol due to the breakage of intra-chain H-bonds which participate in the stiffening of the macromolecular structure.

*Even if the assumption of a unique master curve for all Gx formulations is not totally justified, it remains that the overlap volume fraction of the mixtures ($\phi_x^*$) can only be higher than the extrapolated one, which in this case would correspond to an even greater deswelling of the agar chain.*

## 6. Water/Glycerol agar gels

*6.1. Gelation and mechanical properties*

As shown in **Table S2**, the viscoelastic data are in good agreement with the thermodynamic analysis carried out by DSC with a good correlation between the association temperatures of agar mixtures ($T_{as}$). The transition temperature changes very little in the range of 40 °C for glycerol fractions between 0 and 40 wt%, and then decreases significantly for 60, 80 and 100 wt%. This can be attributed to the increasing viscosity of the medium that slows down the self-assembly of agar. The apparently high value of $T_{sol\text{-}gel}$ observed for G100 compared to G80 is a consequence of the very high viscosity of the solvent, 13 to 22 times higher for G100 compared to G80 between 40 and 20 °C, which proportionally increases the reptation time of entangled polymer chains.

**Table S2**. Characteristic temperatures of association and gelation upon cooling as determined by DSC and rheology.

| $Gel$ | $T_{max}^{DSC}$ (°C) | $T_{as}^{DSC}$ (°C) | $T_{as}^{Rheo}$ (°C) | $T_{sol-gel}^{Rheo}$ $(°C)$ |
|---|---|---|---|---|
| $G0$ | 32.5 | 43.9 | 42.7 | 39.2 |
| $G20$ | 32.2 | 40.8 | 42.2 | 39.6 |
| $G40$ | 31.8 | 37.2 | 40.6 | 38.7 |
| $G60$ | 23.2 | 31.3 | 36.1 | 34.7 |
| $G80$ | 15.6 | 24.1 | 29.7 | 26.5 |
| $G100$ | | | 28.2 | 36.5 |

By considering the agar volume fraction ($\phi_{agar}$) as the relevant descriptor of the formulations, rather than the mass fraction which is the same for all (3.6 wt%), we can see in **Figure S8** that the Young's modulus of G/W formulations follow the same trend ($E \sim \phi^{2.1}$) as previously determined with agar hydrogels. This demonstrates that the apparent increase in elastic modulus with glycerol content, from G0 to G80, arises primarily from the relative increase in agar volume fraction. On the other hand, the properties of agar gels formulated in pure glycerol (G100) remains well below the master curve in relation with the formation of a very weak network.

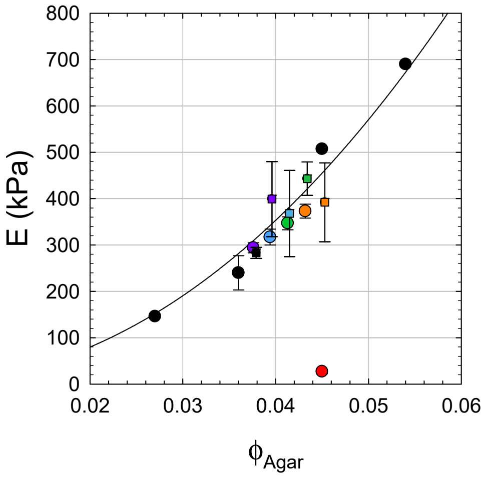


**Figure S8**

Variation of the Young's modulus as a function of the agar volume fraction in the Gx formulations without talc (● G0, ● G20, ● G40, ● G60, ● G80, ● G100) and with talc (■ G0$^T$, ■ G20$^T$, ■ G40$^T$, ■ G60$^T$, ■ G80$^T$). The black circles correspond to mechanical tests performed with agar gels prepared with different volume fraction in pure water and the solid line is the scaling relation obtained in water: $E \sim \phi^{2.1}$.

The same applies to composite gels (Gx$^T$ in **Figure S8**), which were prepared in the presence of talc particles and studied by rheology and tensile tests (**Figure S9**).

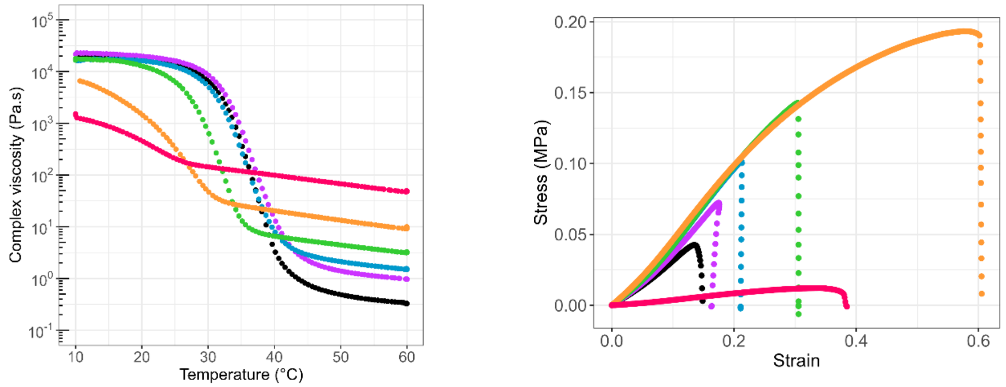


**Figure S9**. Rheological (**left**) and mechanical (**right**) analyses performed on composite agar gels prepared with various amounts of Glycerol/Water co-solvent and fixed agar and talc concentrations, $C_{agar}$ = 3.6 wt% and $C_{talc}$ = 8.3 wt%, respectively. (● G0$^T$, ● G20$^T$, ● G40$^T$, ● G60$^T$, ● G80$^T$, ● G100$^T$)

Once again, this trend highlights the fact that the elastic modulus of mixed gels is essentially controlled by the agar volume fraction, and that talc particles do not interact specifically with the polysaccharide network.

### *6.2. Swelling of agar gels in glycerol*

In contrast to equilibrium data in water, analyses in glycerol are more difficult to perform, due to 1) the high viscosity of glycerol which increases the uncertainty of weighing (difficulty in removing the

surface solvent), and 2) the poor mechanical properties of the gels immersed in glycerol which can also lead to dimensional variations when removed from their baths (**Figure S10**).

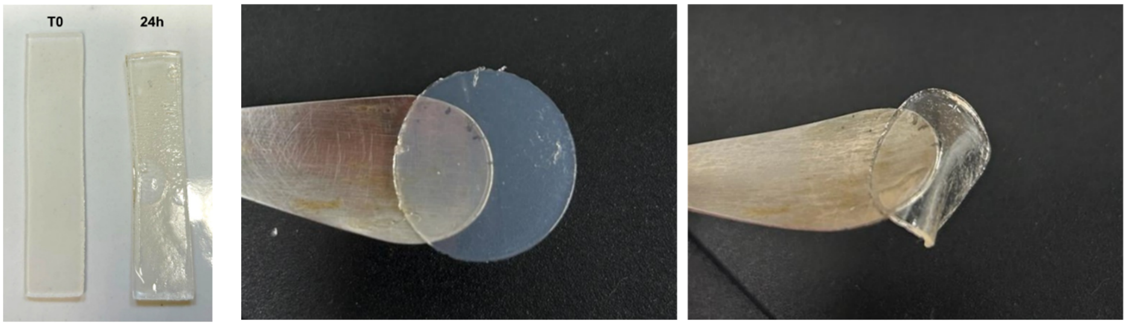


**Figure S10. Left**: Photo of a G20 strap before (**T0**) and after **24** hours immersion in glycerol. **Middle**: Photo of a G0 disc before swelling (as prepared). **Right**: Photo of the same G0 disc after 48h swelling in glycerol.

In order to best interpret the equilibrium data in glycerol, the experimental density variation as a function of time was calculated as : $\rho/\rho_0 = (m/m_0)/((Lw/L_0w_0)^{3/2})$ and the volume change was indirectly determined from the mass ratio assuming total exchange of the initial solvent mixture ($\rho_0$) with glycerol ($\rho_{Gly}$) : $V/V_0 = (m/m_0)/(\rho_{Gly}/\rho_0)$. From the whole set of data (see **Figure S11**), it is interesting to note that solvent exchange occurs much more slowly in glycerol, since a decrease of volumes is observed during the first 4 days before the increase thereafter. Despite the dispersion of density data with time, the results suggest total water elimination after 4 to 8 days of equilibrium. This is confirmed by TGA showing that there is no residual water in the G0 hydrogel after 4 days in glycerol (see **Figure S12**).

This very slow kinetics compared to previous measurements performed in water ($t_{eq}$ < 4 h) can be attributed to a significant decrease in the diffusion coefficients of water and glycerol between the two media considered: the diffusion coefficient of water decreases from 2.3. $10^{-5}$ $cm^2.s^{-1}$ in water to 1.4.$10^{-7}$ $cm^2.s^{-1}$ in glycerol, and that of glycerol decreases from 1.0.$10^{-5}$ $cm^2.s^{-1}$ in water to 2.5.$10^{-8}$ $cm^2.s^{-1}$ in glycerol (4).

$V/V_0$ $\rho/\rho_0$

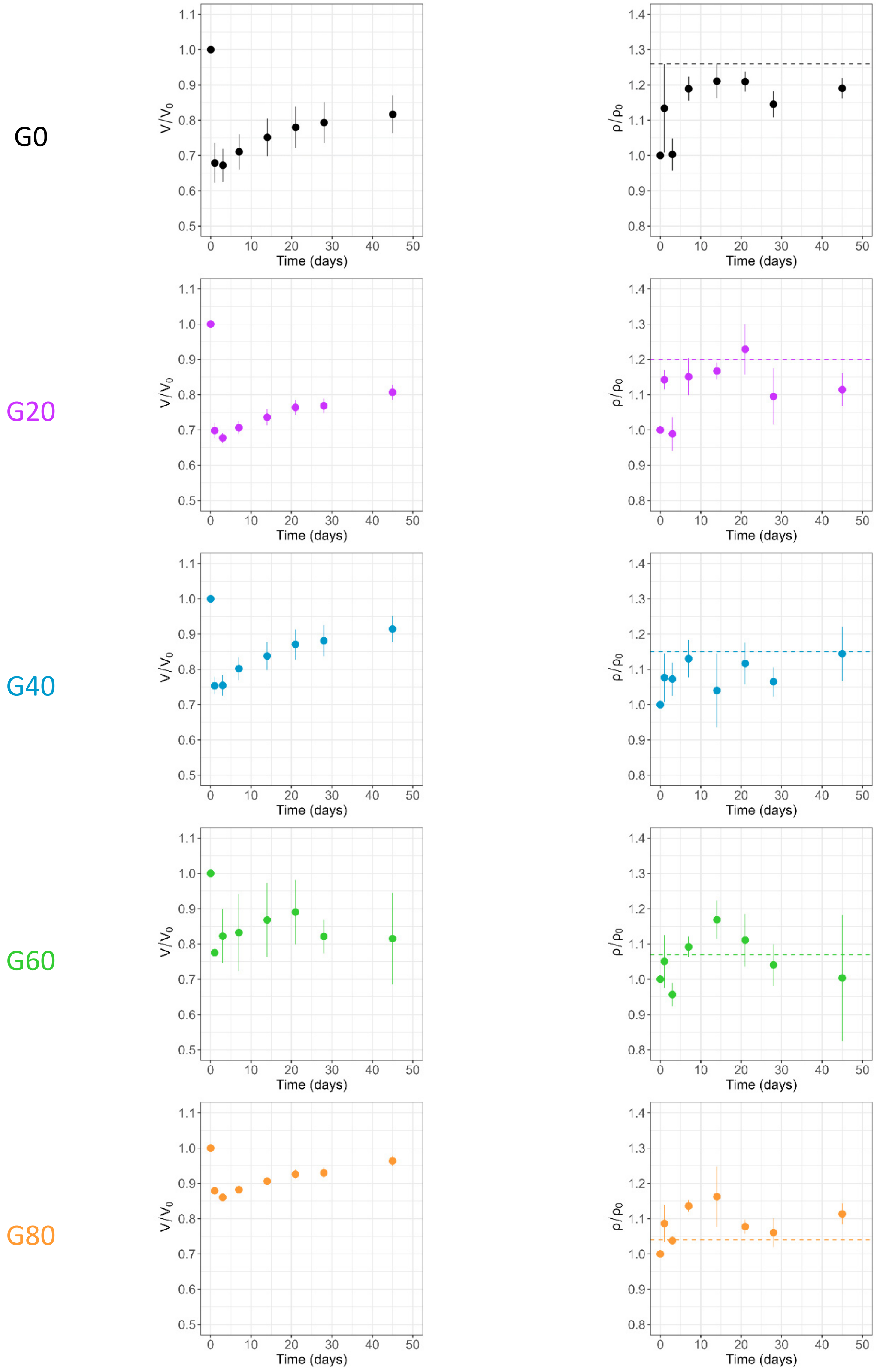


**Figure S11.** Swelling kinetics of glycerol/water agar gels immersed in glycerol for 45 days with $V/V_0$ and $\rho/\rho_0$ the variations of volume and density. The dotted line represents the theoretical variation of density after complete exchange with glycerol.

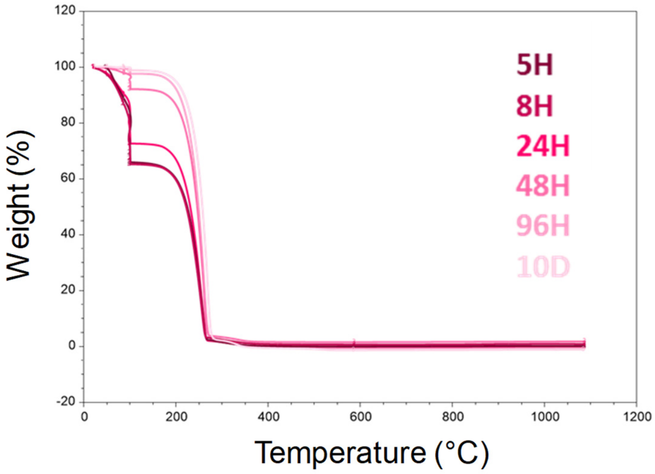

**Figure S12** TGA monitoring of G0 gel during swelling in glycerol.

Assuming that all the water has been released after 4 to 8 days, the calculated change in volume $V/V_0$ based on this assumption can be considered quantitative beyond this period, but remains qualitative for shorter times. Nevertheless, all the studies carried out on the different gels clearly demonstrate an initial deswelling process, corresponding to a much faster outflow of water than the inflow of glycerol, accompanied by a decrease in stiffness and an increase in transparency (see **Figure S10**). These variations can be attributed to a gradual dissociation of helix bundles in smaller domains, leading to a relaxation of the stresses locked during gelation. The higher the initial water content, the greater the decrease of volume. After one week of solvent exchange, the re-swelling of the gels over time is a continuation of the previous process with a slow inflow of glycerol into the gel which seem to reach an equilibrium after 40-50 days. Taking into account the initial volume fraction of agar in the different formulations and the ratio $V/V_0$ at the end of the swelling experiments, one can try to calculate the final volume fraction of agar gels in glycerol which interestingly is almost the same and equal to $\phi_{agar} = (4.50\pm0.15).10^{-2}$ for all of them. Contrary to gelation in water which proceeds through double helices formation and phase separation of these helices in robust bundles forming an energetic network, all the gels swollen in glycerol behave as entropic networks able to reach some equilibrium. In these conditions, the disaggregation of bundles release the constraints initially locked during phase

separation. Eventually, dissociated chains could be extracted from the network, but this hypothesis has not been verified.

*6.3. Aging of agar gels in air*

Aging tests were carried out on gel strips left at room temperature and open air, by measuring their dimensions and mass at different times. Except for G0 hydrogel, which is almost completely dehydrated and severely deformed after 48 h (**Figure S13**), all the gels prepared in glycerol/water mixtures reach equilibrium after 48 h with a mean residual water content of about 15 wt% (see **Figure S14**).

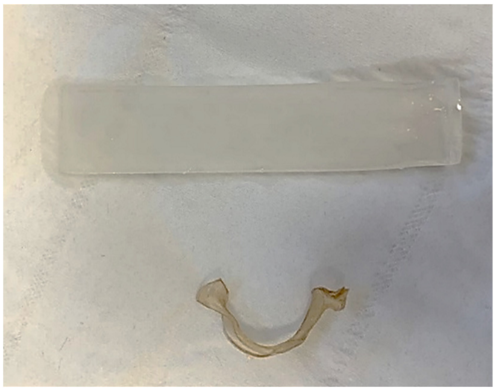

**Figure S13**

Images of G0 hydrogels before (up) and after (down) aging at room temperature and open air.

The antagonistic consequences of air ageing are an increase of the concentration of the agar network, which should enhance mechanical properties, and a similar increase in glycerol content up to 80-90 % by weight, which would on the contrary tend to weaken the network.

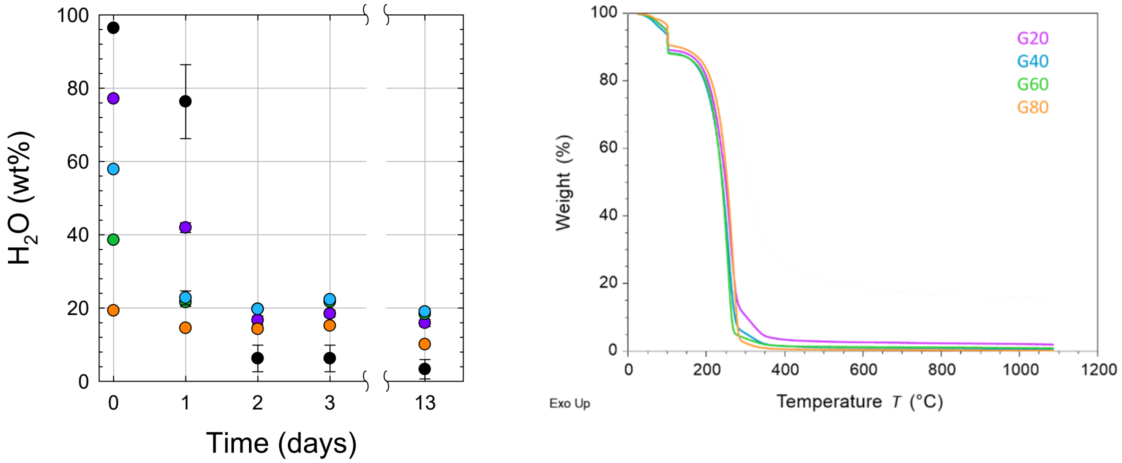


**Figure S14**. **Left**: Kinetics of drying of Gx gels during aging at room temperature and open air (● G0, ● G20, ● G40, ● G60, ● G80).
**Right**: TGA on glycerol/water gels after 48h aging in ambient air and room temperature.

Such behaviour highlighted with Gx gels is also observed for hybrid gels incorporating talc, with increased rigidity for $G20^T$, where the increase in agar concentration prevails over glycerol, and lower moduli for $G60^T$ and $G80^T$ where the high glycerol content outweighs the concentration effect (**Figure S15**).

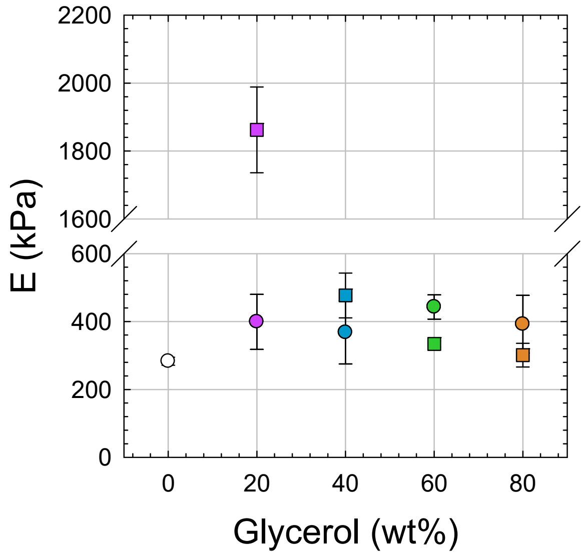


**Figure S15**

Comparison of Young's moduli of glycerol/water gels incorporating talc, before (circles) and after 48h aging (squares).

*6.4. Towards application: analysis of agar gel tips*

Aiming to replace PUR catheter tips used in animal insemination, soft agar gel tips have been molded using fixed concentrations of agar (C= 3.6 wt%) and talc (8.3 wt%) and various weight ratios of glycerol to water.

*6.4.1. Aging in open air*

Aging experiments were carried out on gel tips in similar conditions to those described above for agar strips. Monitoring the mass and height of agar tips as a function of exposure time (see **Figure S16**) shows that they reach equilibrium after around 1 week of exposure to open air, with similar deswelling proportional to the initial water content. After 11 days, the mass variations are 88 % for $G0^T$, 67% for $G20^T$, 45 % for $G40^T$, 28 % for $G60^T$ and 6% for $G80^T$. Similar mass variations were also obtained with accelerated ageing tests performed at 30°C, the equilibrium being reached in only 1 day in these conditions (see **Figure S16**).

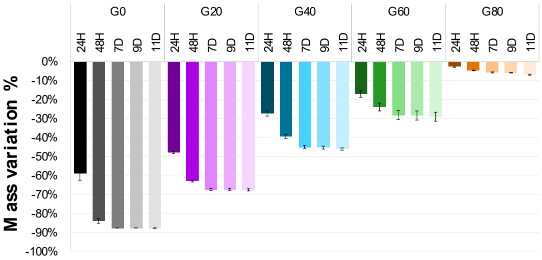


**A**

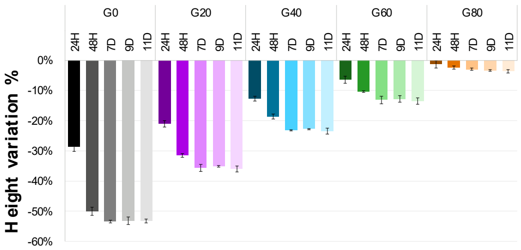


**B**

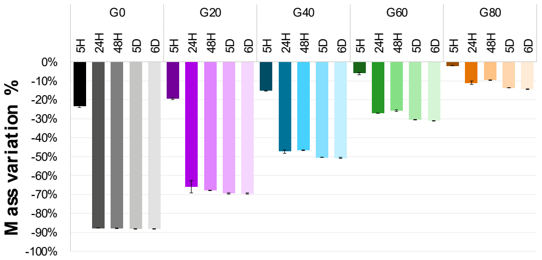


**C**

**Figure S16**
Kinetics of drying at open air and room temperature (mass (**A**) and height (**B**)), and 30 °C (mass (**C**)), of agar gel tips prepared with various weight ratio of glycerol/water ($Gx^T$).
Initial concentrations of agar (C=3.6 wt%) and talc (C=8.3 wt%) were the same for all the tips

Although the equilibrium time of gel tips is much longer than gel strips, due to the bigger size of the objects, the comparison between mass and height demonstrates an isotropic deswelling according to $(V_t/V_0) = (h_t/h_0)^3 = (m_t/m_0).(\rho_0/\rho_t)$ with V, h, m and ρ the volume, height, mass and density at time 0 or t (**Figure S17**).

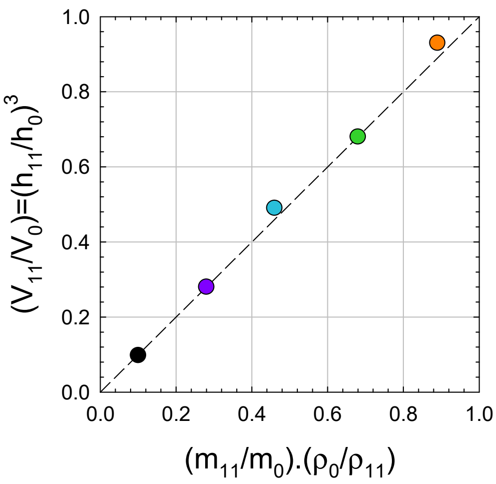


**Figure S17**

Comparison between height and mass analyses performed on agar gel tips prepared with various weight ratio of glycerol/water ($Gx^T$), after 11 days of drying at room temperature in open air:
(● G0, ● G20, ● G40, ● G60, ● G80).
$V_x$, $h_x$, $m_x$ and $\rho_x$ correspond to volumes, heights, masses and densities calculated at time 0 and after 11 days.

### *6.4.2. Mechanical properties: G60$^T$ versus PUR foam*

As shown in **Figure S18**, the mechanical behaviours of the two materials are relatively comparable, although a few differences are worth mentioning.

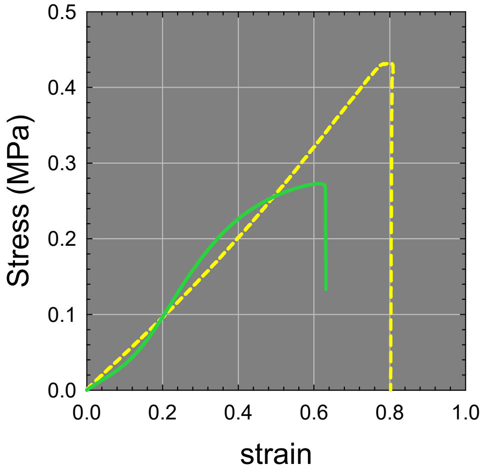


**Figure S18**

Comparative stress-strain curve obtained with PUR foam (yellow dashed line) and $G60^T$ agar gel (solid green line) after aging at room temperature for 48h.
Young's modulus (E) and elongation at break ($\varepsilon_R$) are given below.

| Sample | E (kPa) | $\varepsilon_R$ |
|---|---|---|
| $G60^T$ | 334 ± 9 | 0.62 ± 0.11 |
| PUR foam | 530 ± 80 | 0.73 ± 0.07 |

After 48 hours ageing at room temperature, the $G60^T$ gel has a Young's modulus of 334 kPa, lower than that of PUR foam (E=530 kPa). Nevertheless, while the stress-strain profile of PUR remains linear over the entire deformation range up to failure, $G60^T$ hardens significantly above 10% deformation. For larger deformations, the trend is reversed and $G60^T$ gel becomes less deformable than PUR foam. Regarding the elongation at break, $\varepsilon_R = 0.62$ for $G60^T$ and 0.73 for PUR, we can consider that the two materials are comparable and this can be a general conclusion over the whole set of tensile properties.

### *6.4.3. In vitro toxicity*

As described in the experimental section, tip toxicity was assessed by analysing sperm quality after passage through the insemination probe fitted with a $G60^T$ or PUR tip (see **Figure S4**). As shown in **Figure S19**, the average motility of sperm after 1 day or 6 days of storage before the passage through the probe are 88 % and 84 %, respectively.

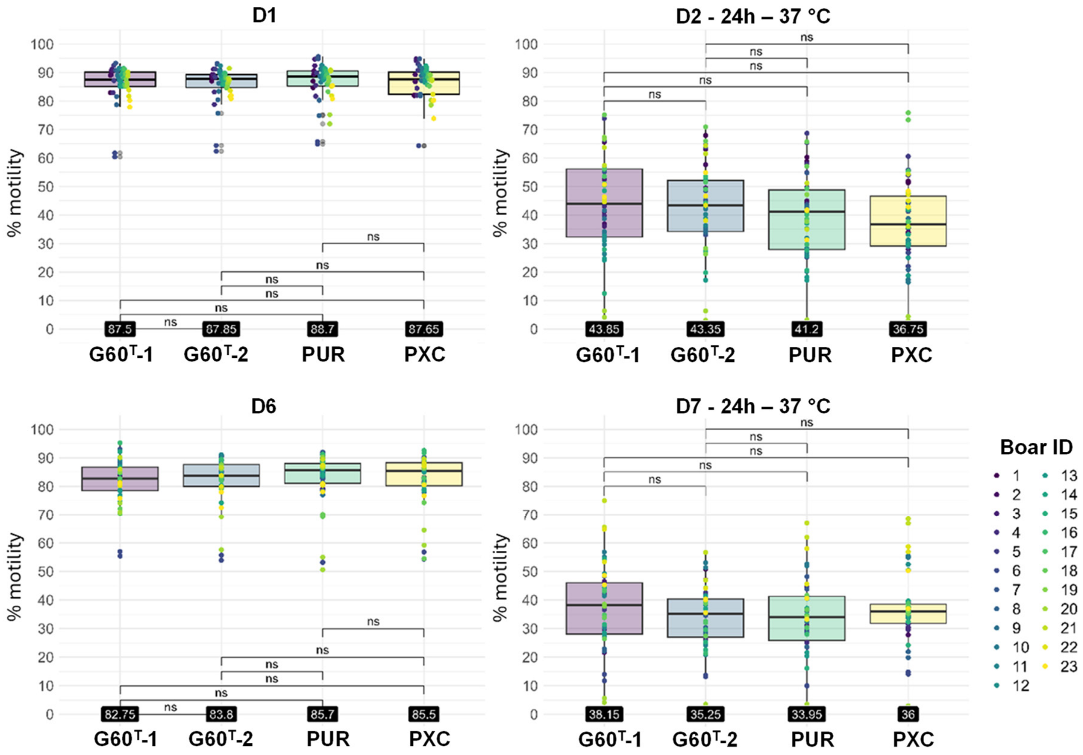


**Figure S19**. Evaluation of sperm motility with IVOS® after 1 day (D1) or 6 days (D6) storage at 17 °C and passage of the semen through the probe (G60$^T$-1, G60$^T$-2 and PUR), followed by 24-hour thermoresistance tests performed at 37 °C (D2 and D7). A control semen solution was analysed under the same conditions (PXC).

These values drop to 41% and 35 %, respectively, after the temperature-resistance test (TRT) performed at 37 °C during 24 hours. Under the conditions tested, based on a mixed linear model with fixed effects (analysis time and conditions) and random effects (boars), these results demonstrate that there is no significant impact of the tip material on sperm motility by comparison with the reference PXC.

As with motility, the viability results (**Figure S20**) do not show any significant difference. The viability was around 92 % after 1 and 5 days of storage, falling to 80 % and 75 %, respectively, after temperature-resistance test.

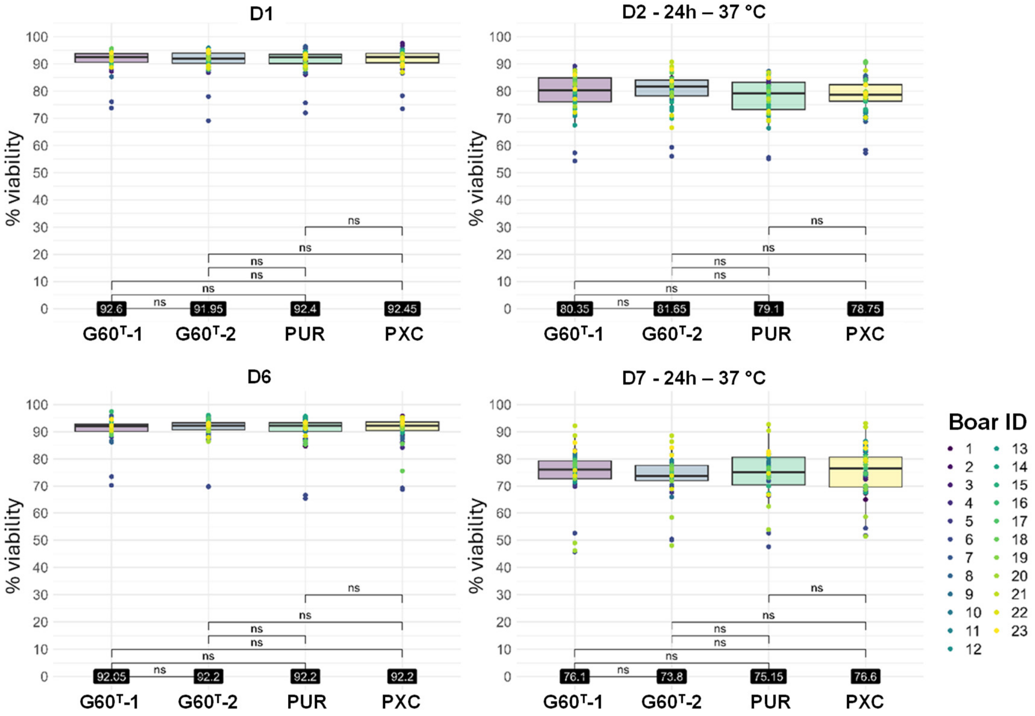


**Figure S20**. Evaluation of sperm viability with IVOS® after 1 day (D1) or 6 days (D6) storage at 17°C and passage of the semen through the probe (G60$^T$-1, G60$^T$-2 and PUR), followed by 24-hour thermoresistance tests performed at 37 °C (D2 and D7). A control semen solution was analysed under the same conditions (PXC).

Finally, a more detailed analysis of acrosomal integrity and mitochondrial potential which allow to differentiate between viable, viable with intact acrosomes, intact acrosomes, mitochondrial potential, confirm the absence of toxicity of both agar and PUR tips on sperm quality (**Figure S21**). Indeed, the total proportion of sperms with intact acrosomes was 95 % and 90 % after 1 and 5 days of storage respectively, falling to 85 % and 75 % after TTR. For the population with intact mitochondrial integrity, the proportion was around 55 % before and after TTR. This whole set of data, confirms that these new materials based on agar, talc, water and glycerol are non-toxic for sperms.

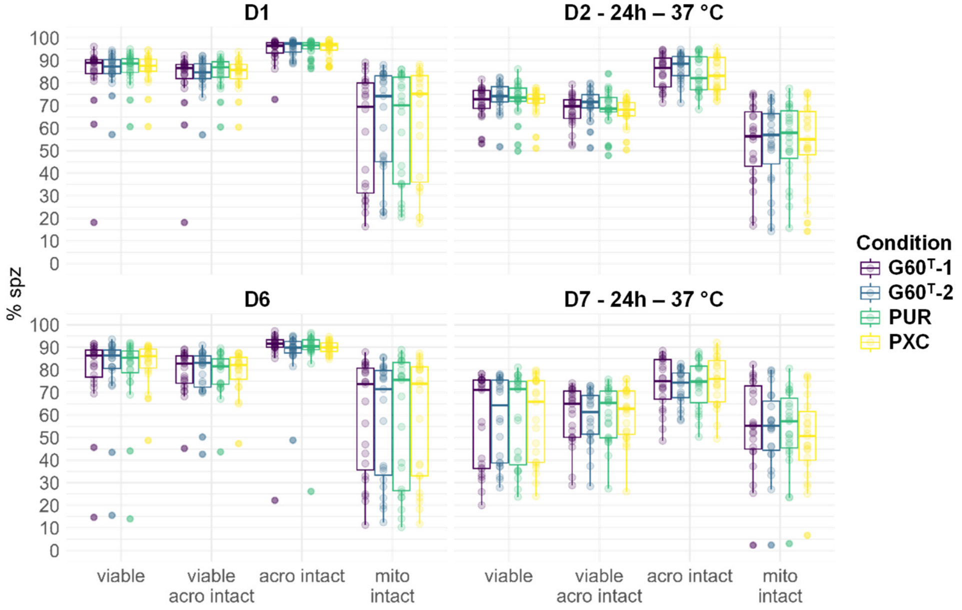


**Figure S21**. Evaluation of acrosomal and mitochondrial integrity with EasyCyte$^{TM}$ after 1 day (D1) or 6 days (D6) storage at 17 °C and passage of the semen through the probe (G60$^{T}$-1, G60$^{T}$-2 and PUR), followed by 24-hour thermoresistance tests performed at 37 °C (D2 and D7).

# References


(1) Langendijk, P.; Soede, N.; Kemp, B. Theriogenology 2005, 63 (2), 500-513.

(2) Willenburg, K.; Miller, G.; Rodriguez-Zas, S.; Knox, R. Journal of Animal Science 2003, 81 (4), 821-829.

(3) Takamura, K.; Fischer, H.; Morrow, N. R. Journal of Petroleum Science and Engineering 2012, (98-99), 50-60.

(4) D'Errico, G.; Ortona, O.; Capuano, F.; Vitagliano, V. Journal of Chemical & Engineering Data 2004, 49, 1665-1670.
